# Parallel Expansions of Sox Transcription Factor Group B Predating the Diversifications of the Arthropods and Jawed Vertebrates

**DOI:** 10.1371/journal.pone.0016570

**Published:** 2011-01-27

**Authors:** Lei Zhong, Dengqiang Wang, Xiaoni Gan, Tong Yang, Shunping He

**Affiliations:** 1 Institute of Hydrobiology, Chinese Academy of Sciences, Wuhan, People's Republic of China; 2 Graduate University of Chinese Academy of Sciences, Beijing, People's Republic of China; University of Konstanz, Germany

## Abstract

Group B of the Sox transcription factor family is crucial in embryo development in the insects and vertebrates. Sox group B, unlike the other Sox groups, has an unusually enlarged functional repertoire in insects, but the timing and mechanism of the expansion of this group were unclear. We collected and analyzed data for Sox group B from 36 species of 12 phyla representing the major metazoan clades, with an emphasis on arthropods, to reconstruct the evolutionary history of *SoxB* in bilaterians and to date the expansion of Sox group B in insects. We found that the genome of the bilaterian last common ancestor probably contained one *SoxB1* and one *SoxB2* gene only and that tandem duplications of *SoxB2* occurred before the arthropod diversification but after the arthropod-nematode divergence, resulting in the basal repertoire of Sox group B in diverse arthropod lineages. The arthropod Sox group B repertoire expanded differently from the vertebrate repertoire, which resulted from genome duplications. The parallel increases in the Sox group B repertoires of the arthropods and vertebrates are consistent with the parallel increases in the complexity and diversification of these two important organismal groups.

## Introduction

Sox (Sry-related high-mobility-group box) group B belongs to the Sox family of proteins, which are transcription factors essential in diverse developmental processes [1], [2], including neurogenesis [3], [4], gonadogenesis [5], and lymphopoiesis [6]. The Sox family was initially identified in relation to the mammalian testis-determining factor, SRY, based on the sequence conservation of the single HMG (high-mobility group) domain, which is a domain of about 79 residues [7] that functions in DNA binding, DNA bending, protein interactions, and nuclear transport [1]. Their interaction with other tissue-specific transcription factors and their spatiotemporal expression patterns, together with mutations in the HMG domain, allow different Sox transcription factors to specify their target selection [1], [8], [9]. After earlier phylogentic analyses on the HMG superfamily involving the Sox family [10], [11], the analysis conducted by Bowles *et al*. (2000) based on the HMG domain sequences and other structural indicators, including intron positions, suggested that the Sox family can be classified into groups A–J [12]: A refers to the Sry proteins restricted to some mammals; B, C, D, E, and F are the major groups expressed by a broad range of metazoan taxa [13], [14]; and G–J are particular lineage-specific proteins. This transcription factor family first emerged in the stem of the metazoa, and the bilaterian last common ancestor (LCA) already contained all the major Sox groups in its genome [13], [14].

Group B Sox proteins play crucial roles in neurogenesis, gonadogenesis, morphogenesis, etc. in vertebrates and insects [1], [2], [15], [16], [17]. Within Sox group B, the division into subgroups B1 and B2 has been proposed based on a full-length protein sequence alignment and the functional roles of the group B proteins in chicken [17] and some other vertebrates [12]. In the vertebrates, members of the same subgroup share high similarity of their full-length protein sequences but no observable similarities with members of the other subgroup in the regions outside the HMG domain and a short C-proximal region of this domain. In terms of function, SoxB1 proteins act as transcriptional activators, whereas SoxB2 proteins play a role as repressors in the chicken [17]. SoxB1- and SoxB2-like proteins have also been identified in bilaterian invertebrates and assigned to the two subgroups based on BLAST searches and tree-based analyses, although less confidently [12]. SoxB1- and SoxB2-like proteins have also been identified in the cnidarians [18] and demosponges [13], [19], although with much less confidence, which implies that the division into subgroups B1 and B2 might have taken place before the demosponges diverged from the eumetazoans [13].

However, there is negligible similarity between the protein sequences of the non-HMG domain regions in the members of different SoxB subgroups as in the members of different Sox groups. Therefore, the tree-based phylogenetic analysis of SoxB proteins is actually restricted to the HMG domain as is the analysis of the whole Sox family. However, although the HMG domain sequence has been demonstrated to be sufficient to group the different Sox groups, this domain inadequately resolves the grouping of the subgroups within Sox group B. On published trees constructed from the sequences of Sox group B and the other Sox groups of the bilaterians, the Sox subgroup B2 almost always (and subgroup B1 sometimes) shows paraphyly [12], [20], [21], [22]. When nonbilaterian SoxB sequences are included in the tree construction, the situation becomes more complicated, because the nonbilaterian sequences are often highly divergent and lineage-specific duplications seem to have occurred [13], [18], [23]. On the tree reported in the paper of Shinzato *et al*. (2008), the previously assigned cnidarian and demosponge SoxB1s and SoxB2s all cluster outside the bilaterian Sox subgroup B1 and B2 representatives, which prompted the suggestions that the partition of group B to subgroups B1 and B2 only occurred in the bilaterians and that both SoxB1 and SoxB2 were generated from a SoxB1-like precursor [23]. However, as the authors noted, the tree may contain bias, so the relationship between subgroups B1 and B2 remains unresolved.

Within the Bilateria, vertebrates such as the human and mouse have several representatives from each major Sox group. In contrast, the bilaterian invertebrates typically have only one family member from each of groups C–F, and two members from group B [2], [12]. This difference is considered to have arisen from genome duplications during the early evolution of the vertebrates [24], [25]. However, the situation is quite different in the Sox group B of the insects, in that the insect genomes contain at least four members of this group [20]. There are discrepancies in the assignment of these members to the B1 or B2 subgroups. The early phylogenetic analysis of the *Drosophila* Sox family put three of the four SoxB members in subgroup B2, and suggested that the additional SoxB2 members might have been produced by recent lineage-specific duplications [12]. However, later studies that included more insect genomes revealed that the basal four-member inventory of Sox group B is conserved, at least in the holometabolous insects, and the previously defined insect SoxB2 members have specific sequence and functional features distinguishable from the vertebrate features, which make it difficult to clarify the orthologies between the SoxB members of the insects and the vertebrates [20], [26]. A model has been suggested for the expansion of Sox group B in the insects [26], in which the previously defined SoxB2 member Dichaete of *Drosophila* is orthologous to the vertebrate SoxB1 members, rather than to the vertebrate SoxB2 members. However, this model seems implausible after our investigation, in which we consider orthology a strictly evolutionary concept.

In published papers, only one single species of the noninsect arthropods, the millipede *Glomeris marginata*, has been investigated in terms of its *Sox* genes, and three SoxB members were found in that species [27]. However, only fragments of the HMG domain of the Sox sequences were obtained and no clear orthologies of the SoxB members have been resolved.

Here, we address the questions that underlie the issues discussed above to achieve a more confident and clear understanding of the evolution of Sox group B. In summary, the questions are as follows: When did the subdivision of Sox group B into subgroups B1 and B2 take place? What is the evolutionary trajectory of the expansion of Sox group B found in the insects? Is this expansion insect specific or did it occur homologously in other arthropods, such as crustaceans, myriapods, and chelicerates, or in even broader taxa? To answer these questions, we collected data from representative metazoan lineages and the metazoans' closest relative, and reconstructed the evolutionary scenario of Sox group B in the metazoans.

## Results and Discussion

### 1. Phylogenetic origin of the Sox subgroup B1/B2

**Figure 1:**
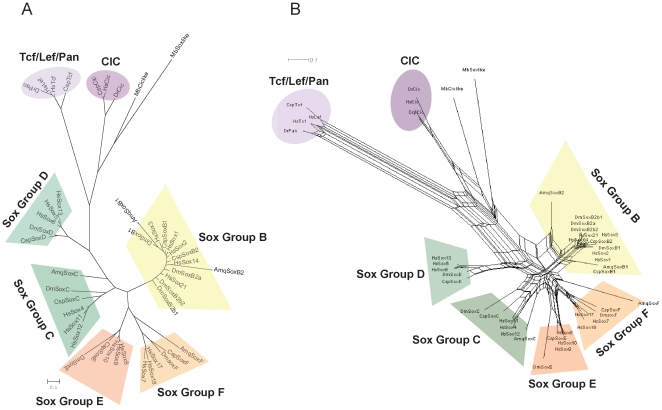
Bayesian tree and split network of representative metazoan proteins based on an HMG domain alignment with Sox, Cic and Tcf/Lef/Pan sequences of the human, *Drosophila*, and annelid, and Sox-like and Cic-like sequences of the choanozoan. (A) Bayesian tree is shown, reconstructed under the RtREV + G (gamma distribution) model. (B) Split network reconstructed under the JTT model. Abbreviations of species names are as in Table 1.

Larroux *et al*. (2008) suggests that the metazoan LCA had one or two proto-SoxB members, and because the genomes of the fungi and choanoflagellates, the closest relatives of metazoans, contain no Sox sequences, SoxB must have originated after the divergence of the metazoan and choanoflagellate lineages [13]. However, King *et al*. (2008) identified a *Sox*-like sequence in the genome of the choanoflagellate *Monosiga brevicollis* using BLAST [28]. But this Sox-like sequence shares only relatively low identities (<40%) with metazoan Sox proteins in the HMG domain in our analysis, which is significantly below the identities (≥46%; Lefebvre *et al*., 2007 [1]) shown by the metazoan Sox proteins in the HMG domain. The choanoflagellate Sox-like sequence clusters with the Capicua (Cic) sequences on the unrooted Bayesian and Maximum likelihood (ML) trees (Fig. 1A; the ML tree is not shown because it has nearly the same topology as the Bayesian tree) reconstructed with the HMG domains of representative metazoan Sox proteins and the choanoflagellate Sox-like protein, together with representative metazoan nonSox HMG box proteins: T-cell factor (TCF)/lymphoid enhancer binding factor (LEF-1)/pangolin (Pan) and Cic proteins, and the choanoflagellate Cic-like protein. Because conflicting and ambiguous phylogenetic signals can be visualized with split networks [29], we reconstructed a split network using the neighbor-net method based on the same data used in the tree-based analyses. On the split network constructed with the same data (Fig. 1B), the conflicting topologies are displayed simultaneously, and the clustering of the choanoflagellate Sox-like protein with the metazoan Sox proteins is observed. However, the choanoflagellate Sox-like protein is outside the metazoan Sox family, even on the split network, so even if this protein is orthologous to the metazoan Sox family, it is not directly orthologous to a specific Sox group of the metazoans. Therefore, the genesis of Sox group B must have occurred after the divergence of the metazoan lineages and the choanoflagellate lineage.

**Table 1:** Relevant Information for the Species in This Study.

PhylumSubphylumClass	Species	Abbreviation	Number of *SoxB* genes†		Known *SoxB2*/*B1* gene neighborhood
			*B2*	*B1*	
Arthropoda					
Pancrustacea					
Insecta	*Drosophila melanogaster*	Dm	3	1	Chr.3L: *SoxB2b1*, *SoxB2b2*, *SoxB2a*
	*Drosophila mojavensis*	Dmo	3	1	
	*Anopheles gambiae*	Ag	3	1	Chr.3: *SoxB2b1*, *SoxB2b2*, *SoxB2a*, *SoxB1*
	*Tribolium castaneum*	Tc	4	1	LG.5: *SoxB2b1b*, *SoxB2b1a*, *SoxB2b2*, *SoxB2a*, *SoxB1*
	*Apis mellifera*	Am	3	1	LG.11: *SoxB2b1*, *SoxB2b2*, *SoxB2a*
	*Nasonia vitripennis*	Nv	3	1	
	*Pediculus humanus*	Ph	3	1	
Branchiopoda	*Daphnia pulex*	Dp	3	1	Scaff.1: *SoxB2b1*, *SoxB2b2*, *Sox2a*
Malacostraca	*Macrobrachium nipponense* *	Mn	*2*	*1*	
Myriapoda					
Chilopoda	*Mecistocephalus* sp.*	Msp	*2(1)*	*1*	
Diplopoda	*Glomeris marginata*	Gm	*2*	*1*	
Chelicerata	*Araneus ventricosus* *	Av	*4(1)*	*2*	
	*Ixodes scapularis*	Is	3	1	
Tardigrada	*Macrobiotus areolatus* *	Ma	*1(5)*	*1*	
Nematoda	*Brugia malayi*	Bm	1	1	
	*Caenorhabditis elegans*	Ce	1	1	Chr.X: *SoxB2*, *SoxB1*
	*Pristionchus pacificus*	Pp	1	1	
Annelida	*Capitella* sp. I	Csp	1	1	
Mollusca	*Lottia gigantea*	Lg	1	1	Scaff.10: *SoxB2*, *SoxB1*
Platyhelminthes	*Schmidtea mediterranea*	Sm	2	1	
Hemichordata	*Saccoglossus kowalevskii*	Sk	1	2	
	*Ptychodera flava*	Pf	*1*	*1*	
Echinodermata	*Strongylocentrotus purpuratus*	Sp	1	1	Contig NW_001345411.1: *SoxB2*, *SoxB1*
Chordata					
Cephalochordata	*Branchiostoma floridae*	Bf	1	3‡	Scaff.Bf_V2_196: *SoxB2*, *SoxB1c*; Scaff.Bf_V2_196: *SoxB1a*, *SoxB1b*
Tunicata	*Ciona intestinalis*	Ci	1	1	
Vertebrata					
Agnatha	*Petromyzon marinus*	Pm	*1*	*1*	
Chondrichthyes	*Callorhinchus milii*	Cm	*2*	*2*	
Osteichthyes	*Takifugu rubripes*	Tr	3	5	
Amphibia	*Xenopus tropicalis*	Xt	2	3	
Aves	*Gallus gallus*	Gg	2	3	Chr.1: *Sox21*, *Sox1*; Chr.9: *Sox14*, *Sox2*
Mammalia	*Homo sapiens*	Hs	2	3	Chr.13: *Sox21*, *Sox1*; Chr.3: *Sox14*, *Sox2*
Cnidaria					
Hydrozoa	*Hydra magnipapillata*	Hm	3	1	
Anthozoa	*Acropora millepora*	Ami	*2*	*1*	
	*Nematostella vectensis*	Nve	5	1	
Placozoa	*Trichoplax adherens*	Ta	2	1	
Porifera	*Amphimedon queenslandica*	Amq	1	1	
Choanozoa	*Monosiga brevicollis*	Mb	0	0	

NOTE.– Chr., chromosome; LG., linkage group; Scaff., scaffold.

*Species for which the *SoxB* inventory was estimated by sequencing in this study.

† Numerals in normal font indicate that the gene number is based on comprehensive survey in whole genome sequences; numerals in italics indicate that the gene number is based on heuristic survey (by genomic PCR, RT-PCR, or survey in partial genome assembly); the numerals in parentheses indicate the numbers of genes having ambiguous identities because only incomplete HMG box sequences are available for these genes.

‡ One of the three *SoxB1* genes is ambiguous and probably evolved from a *SoxB2* duplicate by gene conversion (discussed in the subsection sf the Results and Discussion).

As mentioned in the Introduction, the subdivision of Sox group B was proposed based on a comparison of the full-length protein sequences and functions of the Sox group B members in chicken [17]. However, the extent to which this subdivision applies should be assessed based on sufficient representative species. We collected and aligned the full-length protein sequences of the Sox group B members of species representing the three major clades (lophotrochozoa, ecdysozoa, and deuterostomia) of bilaterians, nonbilaterian eumetazoans, and basal metazoans, and found that subgroup-specific conservative motifs exist in the region outside the HMG domain in both subgroup B1 [23] and B2 throughout almost all the metazoan representatives, although the conservation becomes less clear when extended to the demosponge SoxB2, and the subgroup-specific conservative motif of subgroup B2 seems to have been lost in some of the protostomes (Fig. S1).

**Figure 2:**
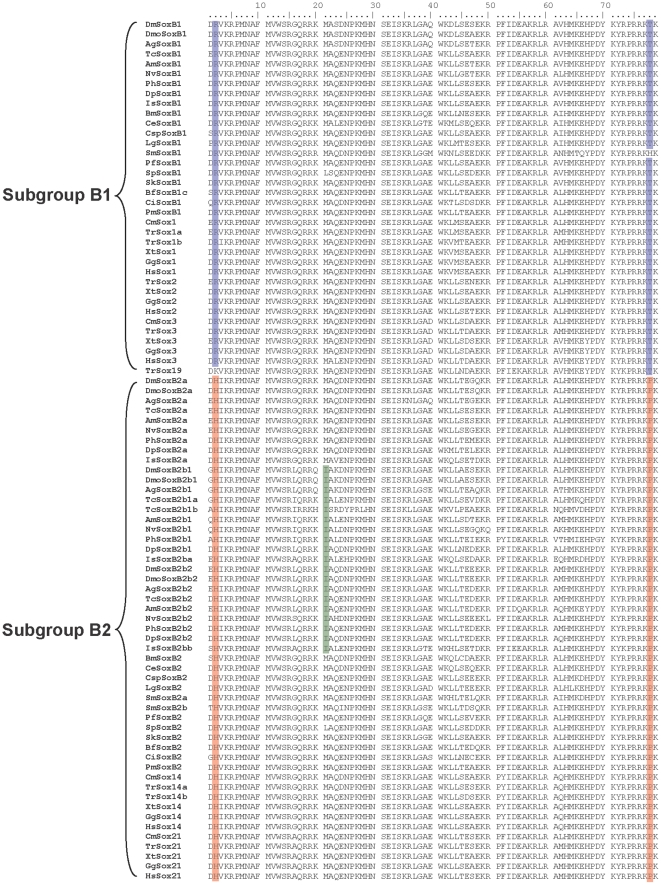
Alignment of the SoxB1/B2 HMG domains from 25 bilaterian species. The signature residues arginine (R) at position 2 and threonine (T) at position 78 for Sox subgroup B1 are shaded in blue; the signature residues histidine (H) at position 2 and proline (P) at position 78 for Sox subgroup B2 are shaded in red; the signature residue isoleucine (I) at position 21 for arthropod SoxB2bs is shaded in green. Abbreviations of species names are as in Table 1.

With a more extensive sampling of the bilaterian SoxB proteins, we first classified the collected SoxB sequences into subgroup B1or B2 according as the best hits of BLASTP searches of the RefSeq protein database of *Homo sapiens*. The alignments of these collected sequences confirmed that the two previously proposed signature residues at positions 2 and 78 of the HMG domain [30], which distinguish Sox subgroups B1 and B2 within group B, are conserved in our much broader sample of taxa, with a few exceptions, which often correspond to highly divergent sequences (Fig. 2). When nonbilaterian SoxBs are included, the signature residues in the HMG domain are incomplete. This reflects either a loss of conservation or suggests that some of the signature residues were derived after the divergence of the bilaterian lineage from the other metazoan lineages. However, in most places, the signature residues histidine (H) at position 2 and proline (P) at position 78 are conserved in the SoxB2 HMG domains of the nonbilaterian metazoans, and the signature residue arginine (R) at position 2 is conserved in the SoxB1 HMG domains of the nonbilaterian eumetazoans (Fig. S2A).

**Figure 3:**
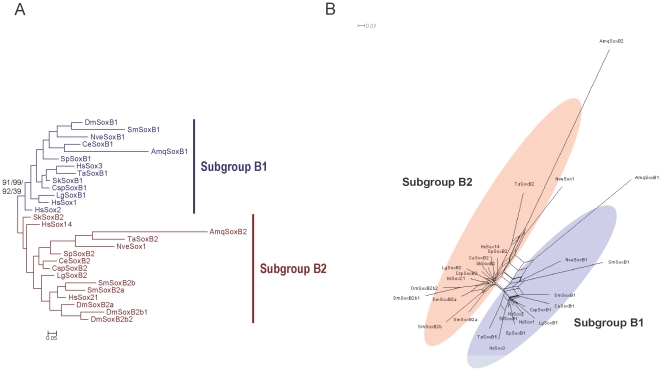
Phylogenetic tree and split network of the representative metazoan SoxB1/B2 proteins based on the HMG domain alignment. (A) Bayesian tree is shown. Statistical support values for the SoxB1/SoxB2 split were derived by different methods: the first number is based on posterior probabilities in the Bayesian analysis; the second number is the support calculated with the approximate likelihood ratio test in the ML analysis; the third number is the support calculated with an SH-like test in the ML analysis; and the fourth number is the support calculated with 100 bootstrap replicates in the ML analysis. The model for the Bayesian reconstruction was RtREV + I (invariable sites) + G; the model for the ML reconstruction was LG + I + G. (B) Split network under the JTT model is shown. Abbreviations of species names are as in Table 1.

We also performed tree-based and net-based phylogenetic analyses of the SoxB HMG domain sequences of species representing 11 phyla of the animal kingdom, excluding some of the highly divergent cnidarian SoxB duplicates. Both the ML and Bayesian trees (Fig. 3A) maintained the split between the SoxB1s and SoxB2s, which was confirmed by the branch supports calculated with the approximate likelihood ratio test and the SH-like test, although the bootstrap value for the ML tree was less than 50%. This low bootstrap value was probably caused by the short length of the sequences (79 residues) and their high identities (>65%), rather than refuting the split of subgroups B1 and B2. Low bootstrap values are prevalent in phylogenetic analyses of the SoxB HMG domains, and these bootstrap values decrease as the number of sequences analyzed increases and/or the length of the sequences decreases [13], [14]. Therefore, the bootstrap test does not seem sufficiently powerful and may be inappropriate for the evaluation of the statistical confidence in the phylogenetic analysis of the SoxB HMG domains. We also reconstructed a split network using the neighbor-net method based on the same data used in the tree-based analyses (Fig. 3B). The split network shows the SoxB1/B2 split and also the possible existence of long-branch attraction (LBA) between the nonbilaterian SoxB1s and SoxB2s, which probably caused the nonbilaterian SoxB1s to be placed outside the bilaterian SoxB1s and SoxB2s in the tree of Shinzato *et al*. (200z8).

Considering the evidence presented above, we can state that the partition of Sox group B to subgroups B1 and B2 makes sense, and reflects the true phylogenetic relationships, and that the SoxB1/B2 division occurred after the divergence of the metazoans and the choanoflagellate but before the demosponges diverged from the eumetazoan lineages.

### 2. Parallel expansions of Sox group B before the arthropod and jawed vertebrate radiations

Previous studies have suggested two incompatible models for the expansion of Sox group B in *Drosophila* [12], [26]. One of these models places one of the four SoxB members into subgroup B1, and the other three into subgroup B2 [12]. Although there is agreement on the assignment of SoxNeuro (SoxB1) into subgroup B1 and Sox21a (SoxB2a) into subgroup B2, the other model maintains that Dichaete (SoxB2b1) and Sox21b (SoxB2b2) are both co-orthologous to both vertebrate Sox1 and Sox2 rather than to the vertebrate SoxB2 members, and that the Protostome–Deuterostome LCA had a three-member complement of Sox group B proteins [26]. The resolution of this dispute lies in the correct orthology assignments of the *Drosophila* SoxB members with the vertebrate ones, and a valid reconstruction of the ancestral SoxB repertoire at key phylogenetic nodes. As we mentioned in the Introduction, a related and interesting question concerns the phylogenetic timing of the expansion of Sox group B in *Drosophila*. Initially, this expansion was attributed to relatively recent duplications [12], but later research [20], [26] involving more insect taxa indicated that the four-member SoxB inventory is phylogenetically old, and was at least present in the LCA of the Hymenoptera and Diptera. However, whether this expansion is even older remained an open question at that time.

To resolve these linked questions, we based our research on an extensive sample of taxa derived from database searches, text mining, and DNA sequencing, which involved 31 species from nine major phyla of bilaterians and five species from three major phyla of nonbilaterian metazoans (Table 1). To represent the major taxonomic groups of Arthropoda, because the arthropods were the focus of this study, our first phylogenetic analysis contained complete data for the Sox group B of eight insects and one branchiopod (subphylum Pancrustacea), and one arachnid (subphylum Chelicerata), and the subsequent analysis added partial data for the Sox group B (Fig. S2B): we retrieved the incomplete HMG domain sequences of one diplopod (subphylum Myriapoda) from the text [27], and newly obtained sequences of one malacostracan (subphylum Pancrustacea), one chilopod (subphylum Myriapoda) and another arachnid (subphylum Chelicerata) by degenerate PCR and genome walking technique; *SoxB* sequences of the microscopic tardigrade *Macrobiotus areolatus*, which belongs to the superphylum Panarthropoda, were also obtained by our *de novo* sequencing, giving the first records of *Sox* genes for the mysterious phylum Tardigrada. In preliminary analyses of the incomplete HMG box sequences we newly determined, some of the sequences show ambiguous orthology (Table 1) and they were excluded from the subsequent phylogenetic reconstructions. Because the nonbilaterian Sox sequences are typically highly divergent, as revealed by previous studies and also our preliminary analyses, we excluded nonbilaterian sequences from the phylogenetic analysis undertaken to resolve the *SoxB* duplication within the Bilateria, to lessen the effects of LBA.

**Figure 4:**
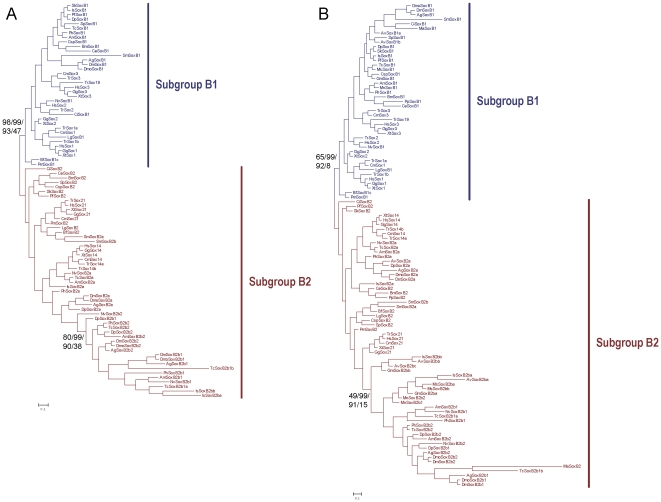
Phylogenetic trees of the bilaterian SoxB1/B2 proteins based on the HMG domain alignment. (A) Bayesian tree based on the alignment in Fig. 2 is shown. Statistical support values for the SoxB1/SoxB2 split and arthropod SoxB2b clade were derived with different methods, as described in Fig. 3. The model for the Bayesian reconstruction was JTT + I + G; the model for the ML reconstruction was LG + I + G. (B) Bayesian tree based on the alignments in Figs. 2 and S2B is shown. Order of statistical support values are as in (A). The model for the Bayesian reconstruction was RtREV + I + G; the model for the ML reconstruction was LG + I + G. Abbreviations of species names are as in Table 1.

**Figure 5:**
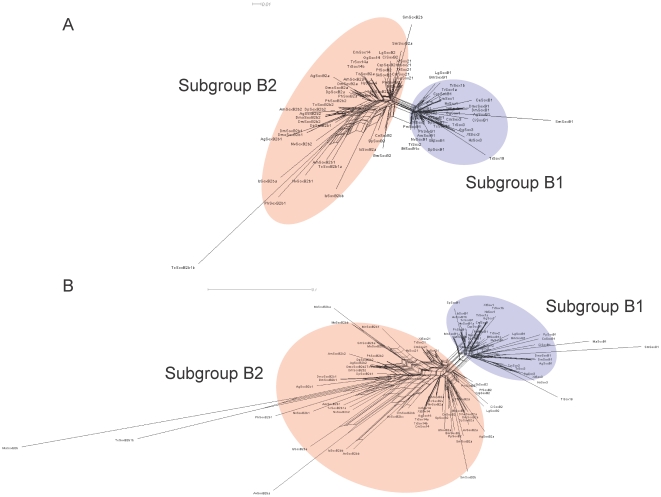
Split networks of the bilaterian SoxB1/B2 proteins based on the HMG domain alignment. (A) Split network based on the alignment in Fig. 2 under the JTT model. (B) Split network based on the alignments in Figs. 2 and S2B under the JTT model. Abbreviations of species names are as in Table 1.

As mentioned above, we first classified the collected SoxB sequences into subgroup B1 or B2 according as the best hits of BLASTP searches of the RefSeq protein database of *Homo sapiens*. The subgroup-specific residues at positions 2 and 78 of the HMG domain [30] are well conserved in the full-length HMG domain alignment of our broad sample of taxa, with only a few exceptions, and none of the exceptions occurs in the arthropods (Fig. 2). We then constructed both tree- and net-based phylogenies based on the alignment in Fig. 2, which contains all the full-length HMG domain sequences of SoxB from the bilaterian species for which the full SoxB complements were available, except three divergent sequences from *Branchiostoma floridae* and *Saccoglossus kowalevskii* (discussed in subsection 4). The ML and Bayesian trees and the split network (Figs. 4A and 5A) all maintained the split between Sox subgroup B1 and subgroup B2, giving support to the previous classification, although the bootstrap value on the ML tree was low, probably because the sequences are short, have high similarity, and there are large numbers of sequences. The high similarity between the subgroup B1 and B2 HMG domains reflects the fact that the net difference between the HMG domains of subgroups B1 and B2, calculated based on the sequences in the alignment (Fig. 2), is 0.04 using p-distances, which corresponds to about three amino acid residues in the 79-residue HMG domain. As discussed in subsection 1, the bootstrap test may be inappropriate for evaluating the statistical confidence at the nodes of the trees constructed for the SoxB HMG domains.

**Figure 6:**
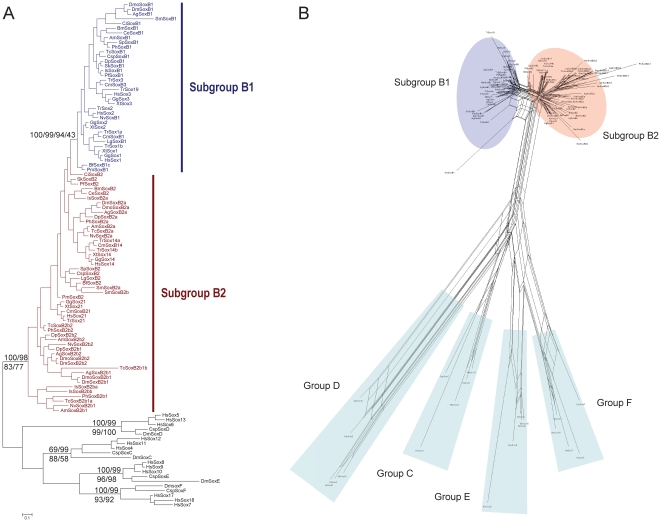
Phylogenetic tree and split network of the bilaterian SoxB1/B2 proteins based on the HMG domain alignment with sequences from Sox groups C, D, E, and F of the human, *Drosophila*, and annelid. (A) Bayesian tree is shown. Statistical support values for the important nodes were derived with different methods, as described in Fig. 3. The model for the Bayesian reconstruction was RtREV + I + G; the model for the ML reconstruction was LG + I + G. (B) Split network based on the data used in (A) excluding TcSoxB2b1a, TcSoxB2b1b, IsSoxB2ba, and IsSoxB2bb, under the JTT model. Abbreviations of species names are as in Table 1.

When the Sox sequences of the other four major Sox groups (C, D, E, and F) of *Drosophila melanogaster*, *Capitella* sp. I, and *Homo sapiens* were used as the outgroups, the clade of subgroup B1 was maintained, but subgroup B2 showed paraphyly in the reconstructed phylogenetic trees and network (Figs. 6A and S3), as in the trees of previously published papers [12], [13], [20]. The paraphyly of Sox subgroup B2 can be explained as a combination of several effects: the metazoan lineages diverged before any large divergence occurred between SoxB1 and SoxB2 in the HMG domain, which caused the internal branch separating Sox subgroup B1 and B2 to be short; SoxB2 experienced fewer functional constraints than did SoxB1 [25], causing more lineage-specific substitutions to accumulate in SoxB2, and SoxB2 displayed nonhomogeneous sequence evolution in different lineages and also between the duplicates generated by multiple lineage-specific duplications, which together resulted in a combination of long and short branches, causing LBA [31]. Even now, the monophyly of subgroup B2 is supported to some extent by the split network (Fig. 6B) from which were excluded four arthropod SoxB2 sequences that formed long branches in the previous network (Fig. S3). Therefore, the nonmonophyly of Sox subgroup B2 in the reconstructed trees probably reflects the influence of statistical errors, such as LBA, rather than refutes the monophyly of Sox subgroup B2.

A systematic nomenclature was developed to better reflect the orthologies between the arthropod subgroup B2 *Sox* genes (Figs. 2 and S2B, and Table 2). The BLAST best matches, phylogenetic groupings (Figs. 4A and 5A), signature residues at positions 2 and 78 of the HMG domain (Fig. 2), and the preservation of the conserved SoxB2-specific non-HMG motif (Fig. S1B) all indicate that the *Drosophila* Dichaete (SoxB2b1) and Sox21b (SoxB2b2) proteins are true descendants of the ancestral SoxB2. Therefore, contrary to the model of McKimmie *et al*. (2005) [26], insect SoxB2b1 is not actually an orthologue of the vertebrate SoxB1 proteins. The fact that *Dichaete* mutants can be rescued by mouse Sox2 of Sox subgroup B1 [16] is not strong evidence for orthology, because functional equivalence is not a part of the definition of orthology as a strict evolutionary concept [32]. The functional equivalence of *Drosophila* Dichaete (SoxB2b1) and mouse Sox2 may reflect either the retention of the function of the ancestral SoxB or convergent evolution.

**Table 2:** Synonymous Names for Arthropod *SoxB* Genes.

*Species*	*Name used in this study*	*Synonymous name(s)*	*Reference(s)*
*Drosophila melanogaster*	*SoxB1*	*SoxNeuro*	[12], [26]
	*SoxB2a*	*Sox21a, SoxB2.3*	
	*SoxB2b1*	*Dichaete, fish-hook, SoxB2.1*	
	*SoxB2b2*	*Sox21b, SoxB2.2*	
*Anopheles gambiae*	*SoxB1*	*SoxNeuro*	[26]
	*SoxB2a*	*Sox21a*	
	*SoxB2b1*	*Dichate*	
	*SoxB2b2*	*Sox21b*	
*Apis mellifera*	*SoxB1*	*SoxNeuro*	[20], [26]
	*SoxB2a*	*Sox21a, SoxB2*	
	*SoxB2b1*	*Dichaete, Sox21*	
	*SoxB2b2*	*Sox21b*	
*Nasonia vitripennis*	*SoxB2a*	*SoxB2*	[20]
	*SoxB2b1*	*Sox21*	
	*SoxB2b2*	*Sox21b*	
*Tribolium castaneum*	*SoxB2a*	*SoxB2*	[20]
	*SoxB2b1a*	*Dichaete*	
	*SoxB2b1b*	*SoxB3*	
	*SoxB2b2*	*Sox21b*	
*Glomeris marginata*	*SoxB2ba*	*SoxB2*	[27]
	*SoxB2bb*	*SoxB3*	

Because there were no SoxB sequences from noninsect arthropods in their study, McKimmie *et al*. (2005) suggested that, although Sox21a (SoxB2a) retained the ancestral form of SoxB2, Dichaete (SoxB2b1) and SoxB21b (SoxB2b2) might represent an insect-specific group. The phylogenetic trees and networks of our study (Figs. 4 and 5) include a wider range of arthropod taxa and suggest that counterparts of the insect *SoxB2b* genes occur in the genomes of the branchiopod, malacostracan, myriapods, and chelicerates. The SoxB2b proteins of the arthropods form a monophyletic group on the trees and network (when the tardigrade SoxB2 is excluded, discussed below) (Figs. 4 and 5) and the signature residue isoleucine (I) at position 21 of the HMG domain in the insect SoxB2b proteins [26] also occurs in the SoxB2b proteins of the noninsect arthropods (Figs. 2 and S2B). None of the SoxB2 proteins in the alignments, other than the arthropod SoxB2b proteins, has isoleucine at position 21, which implies that isoleucine at position 21 is a synapomorphy of the arthropod SoxB2b proteins.

Our preliminary analyses indicated that BLAST methods and tree/net-based methods were incapable of fully deciding the clear orthologies of the SoxB2bs of different insects or those of the insects and noninsect arthropods. However, the conserved gene neighborhoods (CGNs) and conserved intron positions of the *SoxB2* genes of the insects and *Daphnia pulex* allowed the assignment of gene orthologies (Table 1 and Fig. 2). The genome of the shrimp *Macrobrachium nipponense* also contains one intronless *SoxB2b* gene and one *SoxB2b* gene containing an intron at the position conserved among the *SoxB2b2* genes of *Daphnia* and the insects, which indicates that *SoxB2b1* and *SoxB2b2* were present in the genome of the insect–malacostracan LCA. The direct orthologies between the SoxB2bs of the chelicerate *Ixodes scapularis* and the insect SoxB2bs are less clear because there is no intron in the *SoxB* HMG boxes of *Ixodes scapularis* and data on the CGNs of this species are not yet available. However, there is a conserved signature that might distinguish the two SoxB2b paralogues in the protein sequences of the regions outside the HMG domain (Fig. S1B). Because the available sequences are incomplete and there is also no intron in the *SoxB2b* genes of the other chelicerate or the myriapods, no clear orthology assignment among these *SoxB2b* genes is possible at present. However, the myriapods and chelicerates have two or more *SoxB2b* genes, like the insects and crustaceans (Figs. 2 and S2B), so it is highly probable that the arthropod LCA had two *SoxB2b* genes.

The tardigrade *Macrobiotus areolatus*, which is not an arthropod but belongs to the superphylum Panarthropoda, seems to have an enormously large repertoire of SoxB (Table 1). This large repertoire is hardly attributed to the possible contamination of the genomic DNA by the other organisms in environment such as mosses, fungi or bacteria although the tardigrade samples are microscopic, because mosses, fungi and bacteria have no *Sox* gene in their genome, and the obtained tardigrade *SoxB* sequences show particularity when compared with the *SoxB* sequences of the species from the other taxonomic groups by BLAST searches and phylogenetic analyses. The full-length HMG domain sequence of a tardigrade SoxB2 was newly determined by us, but this sequence is highly divergent, and nested in the arthropod SoxB2b clade in the trees and network (Figs. 4B and 5B). Currently, it cannot be clarified whether the SoxB duplicates of the tardigrade share common ancestry of duplication with the SoxB duplicates of the arthropods due to the incompleteness and/or high level of divergence of the tardigrade sequences which causing clear orthology assignments impossible, however, the ongoing genome project of the tardigrade *Hypsibius dujardini* [33] will shed light on this issue.

**Figure 7:**
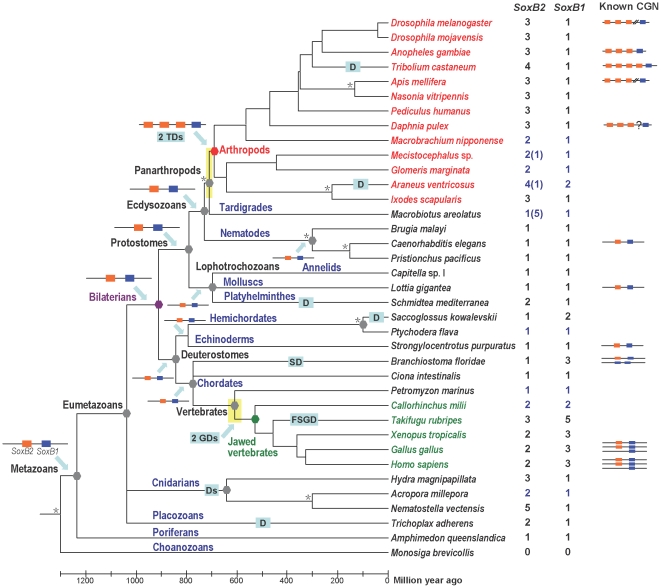
*SoxB1/B2* evolution mapped onto the metazoan timetree. The genes of *Sox* subgroups B1 and B2 are represented by blue and red rectangles, respectively. Numbers on the right side of the tree indicate the estimated inventory of the *Sox* subgroups B2 and B1 respectively in the taxon. The numbers in black indicate that the inventory estimate for the taxon is based on the comprehensive survey in whole genome sequences; the numbers in blue indicate that the inventory estimate for that taxon is based on heuristic survey; the numerals in parentheses indicate the numbers of genes having ambiguous identities because only incomplete HMG box sequences are available for these genes. The known conserved gene neighborhood (CGN) of the group B *Sox* genes in species belonging to that taxon are indicated on the right of the taxon name. The line breaks in *Drosophila* and *Apis* indicate linkage breaks. The question mark in *Daphnia* indicates that the linkage between the *SoxB2* genes and *SoxB1* is not yet determined. The names of phylogenetic groups are indicated beside the nodes or along the terminal branches. The ancestral genomic states of *SoxB1/B2* at the major nodes of the metazoan phylogeny are reconstructed on the principle of parsimony and shown beside the nodes. The two yellow blocks over phylogenetic nodes indicate the range of the phylogenetic timing of the duplication events. The asterisks beside phylogenetic nodes indicate that the timing of the nodes is arbitrary due to lack of information. Abbreviations in rectangles: D, duplication; FSGD, fish-specific genome duplication; GD, genome duplication; SD, segmental duplication; TD, tandem gene duplication.

From the gene inventories and orthology assignments based on the best matches of BLAST, phylogenetic trees and networks, signature residues, conserved intron positions, conserved non-HMG motifs, and the CGNs of Sox group B of the metazoan species examined, we have constructed the evolutionary history of the *SoxB1/B2* genes in the bilaterians. When the gene repertoires and CGNs of *SoxB1/B2* were mapped onto the well-established metazoan phylogeny in the form of a timetree [34], [35], [36], [37], [38], [39], [40] and the ancestral states of *SoxB1/B2* at important phylogenetic nodes were reconstructed based on the principle of parsimony, a picture of the *SoxB1/B2* evolution in the bilaterians emerged (Fig. 7). The bilaterian LCA had one linked pair of *SoxB1* and *SoxB2* genes, which was probably generated by a tandem duplication of the ancestral *SoxB* in the metazoan stem before the demosponge–eumetazoan split. The expansions of *Sox* group B observed in the vertebrates and arthropods occurred later, in mutually independent duplications. During the early evolution of the vertebrates, before the diversification of the jawed vertebrates, two rounds of whole-genome duplication occurred [41], [42], [43], and subsequent gene losses reduced the *Sox* group B repertoire to the complement of three *SoxB1* and two *SoxB2* duplicates we find in the land vertebrates. A third round of genome duplication took place in the stem of the teleost fishes [44], [45], [46], which together with subsequent gene losses, led to the Sox group B repertoires observed in the teleosts [21]. In almost the same period that the vertebrate ancestors underwent their whole-genome duplications, a tandem duplication of *SoxB2* in the genome of the common arthropod ancestor gave rise to *SoxB2a* and the ancestral *SoxB2b*, and a subsequent tandem duplication of *SoxB2b* generated *SoxB2b1* and *SoxB2b2* before the arthropod diversification leading to the extant lineages.

**Figure 8:**
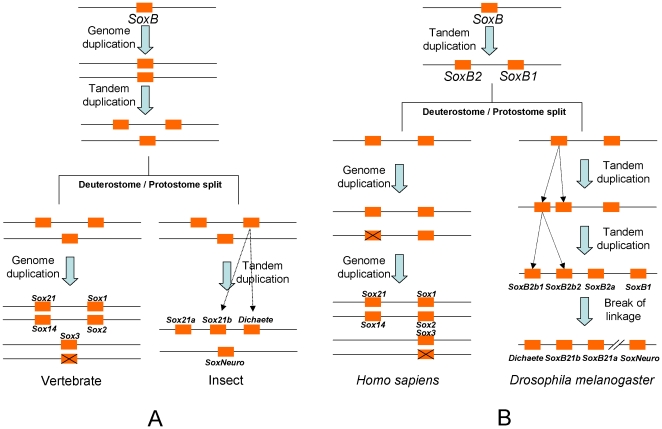
Comparison of the two models of *Sox* group B evolution. (A) The model for *Sox* group B evolution proposed by McKimmie *et al*. (2005) [26]. In this model, an ancestral *SoxB* generate original *Dichaete* and *SoxNeuro* by an ancient genome duplication, a subsequent tandem duplication generate original *Sox21a* before the Deuterostome/Protostome split. After the Deuterostome/Protostome split, a further tandem duplication generated *Sox21b* in insects and an independent genome duplication event increased the copy number of *SoxB* in vertebrates. (B) The model for *Sox* group B evolution proposed in this study. In this model, the Protostome–Deuterostome LCA had one *SoxB1* and one *SoxB2* generated by an ancient tandem duplication of an ancestral *SoxB*. After the Deuterostome/Protostome split, two further tandem duplications gave rise to the additional two copies of *SoxB2* in arthropods, and a linkage break between *SoxB1* and *SoxB2*s occurred in the ancestor of *Drosophila*, resulting in the different chromosome locations of *SoxB1* and *SoxB2*s in *Drosophila*; independently, the vertebrates increased their copy number of *SoxB* through the two rounds of genome duplication. Forks on the rectangles indicate pseudogenization leading to gene loss. *SoxB2b1*, *SoxB2b2*, and *SoxB2a* are the preferred synonyms for *Dichaete*, *Sox21a*, and *Sox21b*, respectively. *Sry* is currently considered to have evolved from allele *Sox3* on the Y chromosome [69], and is therefore not shown in the models.

This scenario of *SoxB* evolution counters the model proposed by McKimmie *et al*. (2005) and adopted by others [25] (Fig. 8A), which suggests that the bilaterian LCA had a total of three Sox group B members. The McKimmie model was perhaps prompted by the false assumptions that *Dichaete* (*SoxB2b1*) is orthologous to vertebrate *Sox2* and that the dislinkage of *SoxB1* and *Dichaete* in *Drosophila* reflects the ancestral state. Because the *SoxB1* and *SoxB2* genes are on one chromosome in the genomes of *Anopheles gambiae* and *Tribolium castaneum* (Table 1), they were probably clustered on one chromosome in the insect LCA. Therefore, the break in the linkage between the *SoxB1* and *SoxB2* genes observed in *Drosophila* must have occurred after the divergence of *Drosophila* from *Anopheles*. A similar linkage break in *Apis mellifera* must have been an independent event. Our model of *SoxB* evolution (Figs. 7 and 8B) also better fits the prevailing hypothesis that two rounds of genome duplication (and a further round in the teleosts) occurred during the evolution of the vertebrates [42].

### 3. Evolutionary significance of the expansion of Sox group B in the arthropods

Gene duplicates can be preserved permanently by neofunctionalization and/or subfunctionalization, thus generating biological novelty and diversity [47]. The lineage-specific expansion of transcription factor families is believed to have played an important role in the increased complexity of animals and in their diversification [48]. Arthropods first appeared near the base of the Cambrian [49] and constitute the most species-rich and ecologically diverse phylum of the animal kingdom. Considering the Sox family's core role in diverse developmental processes [1], [2], [12] and the long-term preservation of the duplicate genes throughout the Arthropoda, the arthropod-specific expansion of the SoxB2 inventory might have provided a versatile genetic tool kit that contributed to the arthropod radiation. Gene duplication provides new genetic material that allows new functions to evolve under relaxed functional constraints and the subfunctionalization of gene duplicates by the divergence of protein expression patterns and/or functions contributes to the establishment of more sophisticated gene networks [47], [50], [51]. Functional studies of SoxB2b1 (Dichaete) in *Drosophila melanogaster* have indicated that this SoxB2 duplicate is crucial to segmentation, neurogenesis, hindgut morphogenesis, cuticle differentiation, and oogenesis [3], [15], [16], [52], [53], [54]. The functional role of *Drosophila* SoxB2b1 in segmentation may be an example of neofunctionalization, because segmentation probably evolved in parallel in the arthropods, chordates, and annelids [55] and the vertebrate SoxB proteins seem to have no such function in embryo development [1]. Interestingly, a similar pattern can be found in the gene *Pax3/7*, the products of which functioned in neurogenesis in the Protostome–Deuterostome LCA but gained a pair-rule function in the common arthropod ancestors [56]. Moreover, species-specific neofunctionalization or subfunctionalization of the *SoxB2* duplicate genes may have taken place, as demonstrated in a comparison of the expression patterns of the orthologous *SoxB2s* of *Apis mellifera* and *Drosophila melanogaster* [20], [26], [57]. The genomic integrity of the *SoxB2* cluster is also retained, at least in insects and *Daphnia*, which diverged over 400 million years ago [40], implying that there are evolutionary constraints on this organization. It will be intriguing to test and compare the functions and organization of the *SoxB2* duplicate genes in species of other arthropod groups.

### 4. Independent duplications of *SoxB* in several other metazoan lineages

In previous studies [25], [58], it was found that the amphioxus *Branchiostoma floridae* has three *SoxB1* genes, which are not directly orthologous to the vertebrate *SoxB1* genes. Our analysis of the amphioxus *SoxB1* genes indicated that amphioxus *SoxB1a* probably evolved from a *SoxB2* duplicate generated by a segmental duplication that produced an additional *SoxB1*/*B2* cluster, and gained the *SoxB1* characteristics through convergent evolution with *SoxB1b*. This suggestion is based on the facts that SoxB2 proteins were the best hits when BLASTP searches were performed in the RefSeq protein database of *Homo sapiens* using the HMG domain of amphioxus SoxB1a as the query; SoxB1a contains the SoxB2 signature residue H at position 2 of the HMG domain, and lacks the conserved SoxB1-specific motifs outside the HMG domain; and the signals for the convergent evolution of amphioxus SoxB1a can be visualized with a split network reconstructed with SoxB sequences of representative bilaterian species (Fig. S4, based on the alignment in Fig. S2C).

In our study, two other bilaterian species provided evidence of the occurrence of a lineage-specific duplication of Sox group B. One species is the platyhelminth *Schmidtea mediterranea*, which contains two paralogous *SoxB2* genes, which may have resulted from a recent duplication. The other species is the hemichordate *Saccoglossus kowalevskii*, which contains an additional divergent *SoxB1* sequence, which has no direct orthologue in other species.

Outside the Bilateria, the anthozoan cnidarian *Nematostella vectensis* has a large repertoire of the Sox family, containing 14 members [18]. Six of the 14 members can be classified into Sox group B but have diverged markedly, and three of them are characterized by an additional residue in the HMG domain [13], [23]. Direct orthologues of some of these divergent *SoxB*s were found in the genome of the hydrozoan cnidarian *Hydra magnipapillata* in our analysis (Fig. S5, based on the alignment in Fig. S2D). Because Anthozoa and Hydrozoa are basal clades of the Cnidaria, these duplications of *SoxB* must have occurred before the cnidarian diversification. Interestingly, the duplications of *SoxB* in the common cnidarian ancestor might have occurred during almost the same period in which the *SoxB* repertoires of the common jawed vertebrate ancestor and the common arthropod ancestor increased in parallel, roughly around 600 million years ago (Fig. 8).

The placozoan *Trichoplax adherens* also has an additional SoxB, with a residue insertion in the same position of the HMG domain as that in the cnidarians (Fig. S2A). It is currently unclear whether this SoxB member emerged before the Placozoan–Cnidarian divergence or independently in the placozoan lineage because the nonbilaterian SoxB sequences are generally divergent and a more extensive sample of taxa is required for its valid resolution.

### Conclusion

We have reconstructed the evolutionary history of the *Sox* subgroups B1 and B2 in the metazoans, reconfirmed that the subdivision of Sox group B into subgroups B1 and B2 took place in the metazoan stem after the metazoan–choanoflagellate divergence but before the demosponge–eumetazoan divergence, and found that after the arthropod–nematode divergence but before the arthropod diversification, the *Sox* subgroup B2 expanded in the common arthropod ancestor to include three members after two successive tandem gene duplications. The bilaterian LCA had only one member from each of the *Sox* subgroups B1 and B2. The *Sox* group B expanded independently in the genomes of the vertebrates and arthropods via different trajectories. This parallel increase in complexity at the molecular level was coincident with parallel increases in complexity and diversification at the organismal level in the vertebrates and arthropods. Functional studies of the Sox subgroup B2 proteins of the arthropods is warranted, and a comparison of the different neofunctionalizations and subfunctionalizations of SoxB2 duplicates in different arthropod groups and between the arthropods and vertebrates should be very interesting and insightful in terms of evolutionary developmental research.

## Materials and Methods

### 1. Data collection

#### 1.1. Data mining

tBLASTN and BLASTP (if the protein database were available) or BLAT searches were performed using the HMG domain of the well-defined Sox group B member SOX1 of *Homo sapiens* as the query with the current assemblies and predicted proteins in the genomes of the examined species to identify and/or retrieve the Sox group B member sequences. The genome databases of the species used for which there was no published study of the Sox family were: the National Center for Biotechnology Information (NCBI) (http://www.ncbi.nlm.nih.gov/) for *Drosophila mojavensis*, *Pediculus humanus*, *Ixodes scapularis*; wFleaBase (http://wfleabase.org/) for *Daphnia pulex*; Wormbase (http://www.wormbase.org/) for *Brugia malayi* and *Pristionchus pacificus*; SmedGD (http://smedgd.neuro.utah.edu/index.html) for *Schmidtea mediterranea*; the UCSC genome browser (http://genome.ucsc.edu/cgi-bin/hgGateway?db=petMar1) for *Petromyzon marinus*; the Elephant Shark Genome Project (http://esharkgenome.imcb.a-star.edu.sg/) for *Callorhinchus milii*; and the Joint Genome Institute (JGI) (http://genome.jgi-psf.org/) for *Xenopus tropicalis*. The genome databases for species for which there were published studies of the Sox family were: NCBI (http://www.ncbi.nlm.nih.gov/) for *Drosophila melanogaster*, *Anopheles gambiae*, *Apis mellifera*, *Tribolium castaneum*, *Caenorhabditis elegans*, *Saccoglossus kowalevskii*, *Strongylocentrotus purpuratus*, *Gallus gallus*, and *Hydra magnipapillata*; and JGI (http://genome.jgi-psf.org/) for *Capitella* sp. I, *Lottia gigantea*, *Branchiostoma floridae*, *Ciona intestinalis*, *Nematostella vectensis*, *Trichoplax adherens*, and *Monosiga brevicollis*. The hits from the searches that showed >45% identity with the whole or almost-whole HMG domain of the query were then used as queries for BLASTX or BLASTP searches in the RefSeq protein database of *Homo sapiens*. A putative SoxB1/B2 member was considered real when its best hit was a formerly defined SoxB1/B2 sequence. The putative SoxB sequences were first classified tentatively into subgroup B1or B2, according to the best hit. The clear orthologies of the group B *Sox* genes were assigned decisively after phylogenetic analysis.

Information on the gene neighborhood of the *SoxB1/B2* genes in *Drosophila melanogaster, Anopheles gambiae, Tribolium castaneum, Apis mellifera, Caenorhabditis elegans, Lottia gigantea, Strongylocentrotus purpuratus, Branchiostoma floridae, Homo sapiens,* and *Gallus gallus* was obtained from the genome databases and the literature [59].

The group B Sox protein sequences for *Homo sapiens*, *Takifugu rubripes*, *Nematostella vectensis*, and *Amphimedon queenslandica* (the Sox protein complements in these four species have genome sequences as background), *Glomeris marginata*, *Ptychodera flava*, and *Acropora millepora*, and Sox21 of *Gallus gallus* were taken from the literature [13], [17], [18], [19], [21], [23], [27], [30], [60]. The Sox-like and Cic-like sequences of *Monosiga brevicollis*, and the Cic, Tcf, and Lef homologues of *Homo sapiens*, *Drosophila melanogaster*, and *Capitella* sp. I were retrieved from NCBI.

#### 1.2. *De novo* sequencing

At the beginning of our study, no genomic sequences for arthropods other than insects and *Daphnia* were available (the genomic sequence of *Ixodes scapularis*, a chelicerate, later became available), so we cloned and sequenced the partial or whole HMG boxes of the group B *Sox* genes of *Macrobrachium nipponense* (De Haan, 1849) (collected from Luzhou, China), *Mecistocephalus* sp., and *Araneus ventricosus* (L. Koch, 1878) (the latter two species were collected from Wuhan, China) to represent the malacostracans, myriapods, and chelicerates, respectively. Since tardigrades, belonging to the superphylum Panarthropoda, are closely related to arthropods, we also cloned and sequenced the partial or whole HMG boxes of the group B *Sox* genes of a tardigrade to represent the microscopic phylum Tardigrada. Individuals of the tardigrade *Macrobiotus areolatus* (Murray, 1907) were extracted from mosses collected from Wudalianchi, China [61], and identified by close examination under a microscope (see Figs. S6, S7, and S8 for the micrographs), then carefully picked out of the sediments by forceps and collected into a centrifuge tube with buffer solution. As templates in the PCR amplifications, genomic DNA was extracted from the fresh tissues of one individual of each given species (*Macrobrachium nipponense*, *Mecistocephalus* sp., and *Araneus ventricosus*) or the whole bodies of several individuals (*Macrobiotus areolatus*, with body lengths of about 500 µm), using a high-salt method. The HMG box fragments of the *Sox* genes were then amplified by PCR using a pair of degenerate primers (5′-ATGAAYGCNTTYATGGTNTGG-3′ and 5′-GGNCGRTAYTTRTARTCNGG-3′) corresponding to the motifs MNAFMVW and PDYKYRP [62], which are found in the HMG domains of almost all Sox proteins belonging to group B. The PCR reactions contained 50 pmol of each primer, 0.1 µg of genomic DNA, 200 µM dNTPs, 1.5 mM MgCl_2_, and 2.5 units of *Taq* DNA polymerase in a 25 µL reaction mix. The PCR amplifications were performed for 35 successive cycles of 95°C for 1 min, 48°C for 1 min, and 72°C for 1 min. The PCR products were resolved on 1.5% agarose gels, and the expected bands were excised and gel purified. The purified products were then subcloned into the pMD18-T vector (TaKaRa Biotechnology [Dalian] Co., Ltd, Dalian, China). The positive clones were sequenced on an ABI 3730 capillary sequencer (Applied Biosystems, Foster City, CA). The Genome Walking Kit (TaKaRa Biotechnology [Dalian] Co., Ltd, Dalian, China) was used to determine the flanking regions of two intron-containing *Sox* products (later shown to be *MnSoxB2b2* and *MaSoxB2*).

The identities of these newly sequenced *Sox* fragments (Genbank accession numbers: FJ805198–FJ805217 and FJ976523) were determined tentatively by BLASTX searches against the RefSeq protein databases of *Homo sapiens* and *Drosophila melanogaster* at NCBI and then by iterative phylogenetic analysis together with the already-defined *Sox* genes. The clear orthologies of these new *Sox* sequences to that of the model species were assigned after extensive phylogenetic analyses. Finally, a revised nomenclature for arthropod *SoxB* genes was developed to better reflect the gene phylogeny.

### 2. Phylogenetic analysis

#### 2.1. Sequence alignment and distance calculations

The full lengths or HMG domains of the collected protein sequences were aligned using ClustalW [63] implemented in the Software MEGA4 [64], and the alignment was adjusted by manual inspection. Pairwise p-distances, within-group mean p-distances and between-groups mean p-distances were computed using MEGA4.

#### 2.2. Phylogenetic reconstruction

ProtTest 2.4 [65] is a program for the selection of the models of protein evolution and was used to determine the models of protein evolution that best fitted our different data sets. ML trees were constructed with the ML method implemented in PhyML 3.0 [66]. Both nearest-neighbor interchange (NNI) and subtree pruning and regrafting (SPR) tree topology searches were used to avoid local optima. The statistical confidence in the nodes was assessed with an approximate likelihood ratio test, which returns χ^2^-based parametric branch support, an SH-like test, and 100 bootstrap replicates. The MrBayes 3.1.2 program [67] was also used to construct the Bayesian trees with the best available model selected by ProtTest 2.4. Two independent Bayesian analyses were run simultaneously for 10 million generations each. Metropolis-coupled Markov chain Monte Carlo with one cold chain and three heated chains was run for each analysis and sampled every 100^th^ generation. A burn-in of 25,000 trees was removed. The convergence of each run was evaluated by plotting the log likelihood value against the number of generations. The statistical confidence in the nodes of the Bayesian trees was evaluated with posterior probabilities. The Software SplitsTree4 [29] was used to generate the split networks using the neighbor-net method [68]. Both p-distances and ML distances under the Jones–Taylor–Thornton (JTT) model were used in this analysis to compare and visualize the conflicting signals.

## Supporting Information

Figure S1 **Alignment of the full-length SoxB1/B2 sequences of representative species.** (A) Alignment of the full SoxB1 sequences of representative species. The yellow line indicates the HMG domain. The blue lines indicate the conservative SoxB1-specific motifs. (B) Alignment of the full SoxB2 sequences of representative species. The yellow line indicates the HMG domain. The red line indicates the conservative SoxB2-specific motif. Abbreviations of species names are as in Table 1.
(PDF)

Figure S2 **Additional alignments of the SoxB HMG domains.** Abbreviations of species names are as in Table 1.
(PDF)

Figure S3 **Split network of the bilaterian SoxB1/B2 proteins based on the HMG domain with sequences from Sox groups C, D, E, and F of human, *Drosophila*, and the annelid** ***Capitella*** **sp. I.** The split network was reconstructed under the JTT model. Abbreviations of species names are as in Table 1.
(PDF)

Figure S4 **Split network of the HMG domain sequences of the SoxB1/B2 proteins in the full complements of** ***Branchiostoma floridae*** **and representative bilaterians, showing the signals of convergent evolution in BfSoxB1a and BfSoxB1b.** The split network is based on the alignment shown in Fig. S2C. Abbreviations of species names are as in Table 1.
(PDF)

Figure S5 **Phylogenetic tree and split network of the HMG domain sequences of the SoxB1/B2 proteins of three cnidarians and representative bilaterians.** (A) Bayesian tree based on the alignment shown in Fig. S2D. Statistical support values for the SoxB1/SoxB2 split and the arthropod SoxB2b clade were derived with different methods, as described in Fig. 3. The model for the Bayesian reconstruction was RtREV + I + G; the model for the ML reconstruction was LG + I + G. (B) Split network under the JTT model is shown. Abbreviations of species names are as in Table 1.
(PDF)

Figure S6 **Photomicrograph of the whole body of the tardigrade** ***Macrobiotus areolatus*** **under a differential interference contrast microscope (DICM) with 100**×** magnification, from the mounting of Tong Yang.**
(JPG)

Figure S7 **Photomicrograph of the pharynx of the tardigrade** ***Macrobiotus areolatus*** **under a DICM with 400**×** magnification, from the mounting of Tong Yang.**
(JPG)

Figure S8 **Photomicrograph of the claws of the tardigrade** ***Macrobiotus areolatus*** **under a DICM with 400**×** magnification, from the mounting of Tong Yang.**
(JPG)
