# Supplementary material for: Parallel Expansions of Sox Transcription Factor Group B Predating the Diversifications of the Arthropods and Jawed Vertebrates
Source: PLoS One. 2011 Jan 27;6(1):e16570. doi: 10.1371/journal.pone.0016570 (PMC3029401; doi:10.1371/journal.pone.0016570)
Supplement: Figure S1 — Alignment of the full-length SoxB1/B2 sequences of representative species. (A) Alignment of the full SoxB1 sequences of representative species. The yellow line indicates the HMG domain. The blue lines indicate the conservative SoxB1-specific motifs. (B) Alignment of the full SoxB2 sequences of representative species. The yellow line indicates the HMG domain. The red line indicates the conservative SoxB2-specific motif. Abbreviations of species names are as in Table 1. (PDF) [file pone.0016570.s001.pdf]

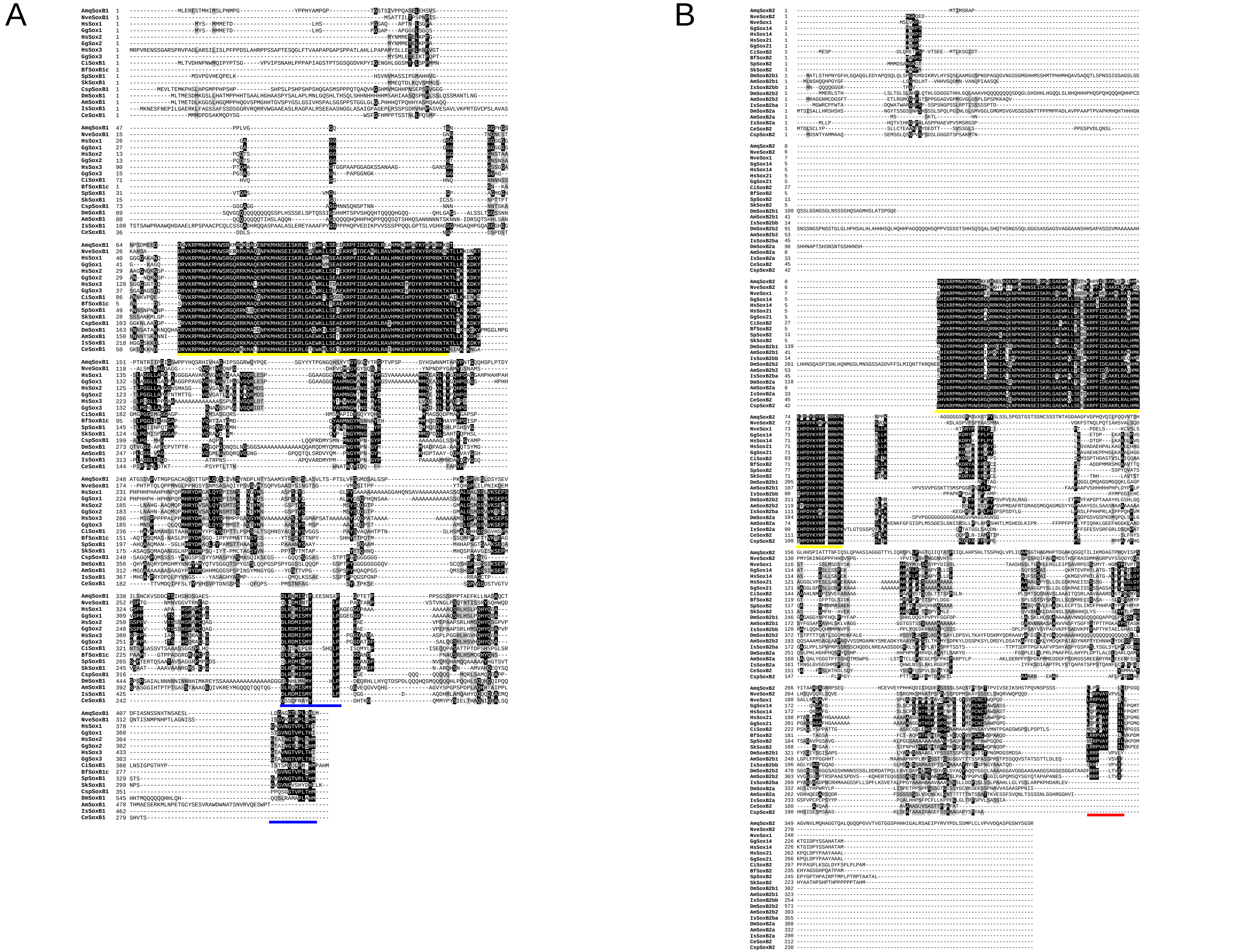
A
B
