# Supplementary material for: Parallel Expansions of Sox Transcription Factor Group B Predating the Diversifications of the Arthropods and Jawed Vertebrates
Source: PLoS One. 2011 Jan 27;6(1):e16570. doi: 10.1371/journal.pone.0016570 (PMC3029401; doi:10.1371/journal.pone.0016570)
Supplement: Figure S2 — Additional alignments of the SoxB HMG domains. Abbreviations of species names are as in Table 1. (PDF) [file pone.0016570.s002.pdf]

A

```
                      10         20         30         40         50         60         70         80
              ....|....| ....|....| ....|....| ....|....| ....|....| ....|....| ....|....| ....|....|
AmqSoxB1      DKVKRPMNAF MVWSRKMRKK IADENPKMHN SEISKRLGTQ WKALSEEDKR PFIDEAKRLR EAHMKKHPNY KYKP-KRKKQ
TaSoxB1       ERVKRPMNAF MVWSRGQRRK MAQENPKMHN SEISKRLGAD WKLLSDADKR PFIDEAKRLR ALHMKEHPDY KYRP-RRKNK
NveSoxB1      DRVKRPMNAF MVWSRERRRK MAQDNPKMHN SEISKRLGSE WKLLSEQEKR PYIDEARRLR AVHMKEHPDY KYRP-RRKSK
AmiSoxB1      DRVKRPMNAF MVWSRERRRR MAQENPKMHN SEISKRLGAE WKQLSDPEKR PYVDEAKRLR AVHMKDHPDY KYRP-RRKSK
HmSoxB1       DKVKRPMNSF MVWSREKRRR LAHENPKMHN SEISKRLGAE WKVLTEDEKA PFVFEAKRLR AEHMKSHPDY KYRP-RRKAK
AmqSoxB2      DHIKRPMNAF MVWSKERRKE LAQENPRMHN SELSKKLGAE WKALSDTNKH RYIEEAKKIR EQHMAEFPHY RYRP-RRKPK
TaSoxB2       DHVKRPMNAF MVWSRVQRRK IAQENPKMHN SEISKRLGAE WKMLSDIEKR PFVDEAKHLR TQHMKDFPDY KYRP-RRKPK
NveSox1       EHVKRPMNAF MVWSREERRK IAQENPKMHN SEISKRLGSE WKQLADDDKK PFVEEAKKLR AQHMKEHPDY KYRP-RRKPK
NveSox2       DHIKRPMNAY MVWSRKERRR IAEECPRMLN SEISKRLGLE WNSLTLDEKQ PYVEEAKRLR ELHKKDHPDY KYQP-KRKPK
TaSoxBa       YHIKRPMNAF IVWSKEQRRK IAEENPKMHN AEISKILGAK WKMLSEGEKR PFINEAKRLH AVHMKIHPEY KYRPRRRKSK
NveSoxB2      GHVKRPMNAF MVWSRGKRKH YASINPRMHN SEISKRLGAE WKMLTAEEKE PFIAEAKRLQ ALHIQEHPDY KYKPKRRKPK
AmiSoxBa      GHIKRPMNAF MVWSRGKRKQ YAAINPRMHN SEISKRLGAE WKMLSQDEKE PFVAEAKRLQ AIHIQEHPDY KYKPKRRKPK
HmSoxBa       SHVKRPMNAF MVWSRGKRRQ MAQDNPRMHN SEISKRLGAE WKCLTQQEKQ PFIDEAKRLR AVHIQEHPDY KYKPKRRKQK
NveSox3       NHVKRPMNAF MVWSKERRRI KSQECPRMHN SEISKILGCE WKATKDELKQ PYIEKAKELQ AQHSRENPGY KYKPRRRKPK
AmiSoxBb      DHIKRPMNAF MVWSKEKRRT MSQKNPKMHN SEISKILGAQ WKKMPDEEKA KYIEEAKRLQ QEHSQKHPDY KYKPRRRKQK
HmSoxBb       DHVKRPMNAF MVWSREKRRK MAQINPRMHN SEISKILGSE WKRMGESEKG PYVLEAKRLQ TQHSIEYPNY KYKPRRRKAK
NveSoxA       QHIKRPMNAF MVWSRTERRK LALKYPNMLN CEISKLLGAE WSRLSEEEKR PFVTEAKRLR TIHNQKYPDY SYKPRRRKSK
HmSoxBc       DHVKRPMNAF MVWSRTERRK LALKYPNMLN CEISKLLGAE WSRMGEEEKS PYIQESKRLR TIHSQKYPDY SYKPRRRKRK
```

B

```
                      10         20         30         40         50         60         70
              ....|....| ....|....| ....|....| ....|....| ....|....| ....|....| ....|....| ....|....
MaSoxB1       ---------- ---SRGQRRK MAQDNPKMHN SAISKCLGAE WKSLNEEDKR PFIDEAKRLR AIHMKEH--- ---------
MnSoxB1       ---------- ---SRGQRRK MAQENPKMHN SEISKRLGAE WKLLSETEKR PFIDEAKRLR AVHMKEH--- ---------
GmSoxB1       ------MNAF MVWSRGQRRK MAQENPKMHN SEISKRLGAE WKLLSEAEKR PFIDEAKRLR AVHMKEHPDY KYRPRRKTK
MspSoxB1      ---------- ---SRGQRRK MAQENPKMHN SEISKRLGAE WKLLSEAEKR PFIDEAKRLR AVHMKEH--- ---------
AvSoxB1a      ---------- ---SRGQRRK MAQDNPKMHN SEISKRLGAE WKLLSEAEKR PFIDEAKRLR AVHMKEH--- ---------
AvSoxB1b      ---------- ---SRGQRRK LAQENPKMHN SEISKRLGAE WKLLSEAEKR PFIDEAKRLR AVHMKEH--- ---------
PpSoxB1       --VKRPMNAF MVWSRGQRRK MAQENPKMHN SEISKRLGTE WKQLQETDKR PFIDEAKRLR QIHMKEHPDY KYRPRRKTK
AvSoxB2a      ---------- ---SRGQRRK MAQDNPKMHN SEISKRLGAE WKQLTELEKR PFIDEAKRLR ALHMKEH--- ---------
MnSoxB2b1     ---------- ---SRMQRRK IAQENPKMHN SEISKRLGSE WKLLTEAEKR PFIDEAKRLR AQHMKEH--- ---------
MnSoxB2b2     DHIKRPMNAF MVWSRMQRRK IAQENPKMHN SEISKRLGAE WKLLTEAEKR PFIDEAKRLR AQHMKDHPDY KYRPRRKPK
GmSoxB2ba     ---------- ---SRAQRRK IAQENPKMHN SEISKRLGAE WKLLTESEKR PFIDEAKRLR AQ-------- ---------
GmSoxB2bb     ---------- ---SRAQRRK IAQENPKMHN SEISKRLGAE WKLLSEAEKR PFIDEAKRLR AV-------- ---------
MspSoxB2ba    ---------- ---SRLQRKK IAQENPKMHN SEISKRLGAE WKLLTEMEKR PFIDEAKRLR AQHMRDH--- ---------
MspSoxB2bb    ---------- ---SRAQRRK IAQENPKMHN SEISKRLGAE WKLLTEMEKR PFIDEAKRLR AQHMRDH--- ---------
AvSoxB2ba     ---------- ---SRAQRRK IAAENPKMHN SEISKRLGSE WKQLSEDQKR PFIDEAKRLR QEHMTLH--- ---------
AvSoxB2bb     ---------- ---SRAQRRK IALENPKMHN SEISKRLGTE WKHLSESEKR PFIEEAKRLR ALHMKEH--- ---------
AvSoxB2bc     ---------- ---SRAQRRK IALENPKMHN SEISKRLGAE WKLLSEAEKR PFIDEAKRLR AVHMKEH--- ---------
MaSoxB2b      KHIKRPMNAF MVWAHERRRQ MSADSPKMHH SNISKILGAE WKLLSAQEKV PFIDEAKKIH TQHMLDHPDY KYRPQRKLK
PpSoxB2       SHVKRPMNAF MVWSRGQRRK MAQDHPKMHN SEISKRLGAE WKMLSETEKR PFIDEAKRLR ALHMKEHPDY KYRPRRKPK
```

C

```
                      10         20         30         40         50         60         70
              ....|....| ....|....| ....|....| ....|....| ....|....| ....|....| ....|....| ....|....
DmSoxB1       DRVKRPMNAF MVWSRGQRRK MASDNPKMHN SEISKRLGAQ WKDLSESEKR PFIDEAKRLR AVHMKEHPDY KYRPRRKTK
CeSoxB1       DRVKRPMNAF MVWSRGQRKK MALENPKMHN SEISKRLGTE WKMLSEQEKR PFIDEAKRLR AIHMKEHPDY KYRPRRKTK
CspSoxB1      SRVKRPMNAF MVWSRGQRRK MAQENPKMHN SEISKRLGAE WKLLSEAEKR PFIDEAKRLR AIHMKEHPDY KYRPRRKTK
LgSoxB1       PRVKRPMNAF MVWSRGQRRK MAQENPKMHN SEISKRLGAE WKLMTESEKR PFIDEAKRLR AIHMKEHPDY KYRPRRKTK
SkSoxB1       DRVKRPMNAF MVWSRGQRRK MAQENPKMHN SEISKRLGAE WKLLSEAEKR PFIDEAKRLR AVHMKEHPDY KYRPRRKTK
SpSoxB1       DRVKRPMNAF MVWSRGQRRK LSQENPKMHN SEISKRLGAE WKLLSEDEKR PFIDEAKRLR AVHMKEHPDY KYRPRRKTK
HsSox1        DRVKRPMNAF MVWSRGQRRK MAQENPKMHN SEISKRLGAE WKVMSEAEKR PFIDEAKRLR ALHMKEHPDY KYRPRRKTK
HsSox2        DRVKRPMNAF MVWSRGQRRK MAQENPKMHN SEISKRLGAE WKLLSETEKR PFIDEAKRLR ALHMKEHPDY KYRPRRKTK
HsSox3        DRVKRPMNAF MVWSRGQRRK MALENPKMHN SEISKRLGAD WKLLTDAEKR PFIDEAKRLR AVHMKEYPDY KYRPRRKTK
DmSoxB2a      DHIKRPMNAF MVWSRGQRRK MAQDNPKMHN SEISKRLGAE WKLLTEGQKR PFIDEAKRLR ALHMKEHPDY KYRPRRKPK
DmSoxB2b1     GHIKRPMNAF MVWSRLQRRQ IAKDNPKMHN SEISKRLGAE WKLLAESEKR PFIDEAKRLR ALHMKEHPDY KYRPRRKPK
DmSoxB2b2     EHIKRPMNAF MVWSRLQRRK IAQDNPKMHN SEISKRLGAE WKLLTEEEKR PFIDEAKRLR AMHMKEHPDY KYRPRRKPK
CeSoxB2       DHVKRPMNAF MVWSRGQRRK MAQDNPKMHN SEISKRLGAE WKQLSEQEKR PFIDEAKRLR ALHMKEHPDY KYRPRRKPK
CspSoxB2      DHVKRPMNAF MVWSRGQRRK MAQENPKMHN SEISKRLGAE WKLLSEEEKR PFIDEAKRLR ALHMKDHPDY KYRPRRKPK
LgSoxB2       DHVKRPMNAF MVWSRGQRRK MAQENPKMHN SEISKRLGAD WKLLTEEEKR PFIDEAKRLR ALHLKEHPDY KYRPRRKPK
SkSoxB2       DHVKRPMNAF MVWSRGQRRK MAQENPKMHN SEISKRLGGE WKLLSEAEKR PFIDEAKRLR ALHMKEHPDY KYRPRRKPK
SpSoxB2       DHVKRPMNAF MVWSRGQRRK LAQENPKMHN SEISKRLGAE WKLLSEDDKR PFIDEAKRLR ALHMKEHPDY KYRPRRKPK
HsSox14       DHIKRPMNAF MVWSRGQRRK MAQENPKMHN SEISKRLGAE WKLLSEAEKR PYIDEAKRLR AQHMKEHPDY KYRPRRKPK
HsSox21       DHVKRPMNAF MVWSRAQRRK MAQENPKMHN SEISKRLGAE WKLLTESEKR PFIDEAKRLR AMHMKEHPDY KYRPRRKPK
BfSoxB1a      DHVKRPMNAF MVWSRIQRRK IAEDNPKMHN SEISRRLGEM WKELSVEEKK PFVEESKRLR AEHMEKYPDY KYRPRRKLK
BfSoxB1b      DRVKRPMNAF MVWSRGQRRK IAEDNPKMHN SEISRRLGEM WKELSVEEKK PFVDESKRLR AKHMEEHPDY KYRPRRKMK
BfSoxB1c      SRVKRPMNAF MVWSRGQRRK MAQENPKMHN SEISKRLGAE WKLLTEAEKR PFIDEAKRLR ALHMKEHPDY KYRPRRKTK
BfSoxB2       DHVKRPMNAF MVWSRGQRRK MAQENPKMHN SEISKRLGAE WKLLTEDQKR PFIDEAKRLR ALHMKEHPDY KYRPRRKPK
```

D

```
                      10         20         30         40         50         60         70         80
              ....|....| ....|....| ....|....| ....|....| ....|....| ....|....| ....|....| ....|....|
DmSoxB1       DRVKRPMNAF MVWSRGQRRK MASDNPKMHN SEISKRLGAQ WKDLSESEKR PFIDEAKRLR AVHMKEHPDY KYRP-RRKTK
CeSoxB1       DRVKRPMNAF MVWSRGQRKK MALENPKMHN SEISKRLGTE WKMLSEQEKR PFIDEAKRLR AIHMKEHPDY KYRP-RRKTK
CspSoxB1      SRVKRPMNAF MVWSRGQRRK MAQENPKMHN SEISKRLGAE WKLLSEAEKR PFIDEAKRLR AIHMKEHPDY KYRP-RRKTK
LgSoxB1       PRVKRPMNAF MVWSRGQRRK MAQENPKMHN SEISKRLGAE WKLMTESEKR PFIDEAKRLR AIHMKEHPDY KYRP-RRKTK
SkSoxB1       DRVKRPMNAF MVWSRGQRRK MAQENPKMHN SEISKRLGAE WKLLSEAEKR PFIDEAKRLR AVHMKEHPDY KYRP-RRKTK
SpSoxB1       DRVKRPMNAF MVWSRGQRRK LSQENPKMHN SEISKRLGAE WKLLSEDEKR PFIDEAKRLR AVHMKEHPDY KYRP-RRKTK
HsSox1        DRVKRPMNAF MVWSRGQRRK MAQENPKMHN SEISKRLGAE WKVMSEAEKR PFIDEAKRLR ALHMKEHPDY KYRP-RRKTK
HsSox2        DRVKRPMNAF MVWSRGQRRK MAQENPKMHN SEISKRLGAE WKLLSETEKR PFIDEAKRLR ALHMKEHPDY KYRP-RRKTK
HsSox3        DRVKRPMNAF MVWSRGQRRK MALENPKMHN SEISKRLGAD WKLLTDAEKR PFIDEAKRLR AVHMKEYPDY KYRP-RRKTK
DmSoxB2a      DHIKRPMNAF MVWSRGQRRK MAQDNPKMHN SEISKRLGAE WKLLTEGQKR PFIDEAKRLR ALHMKEHPDY KYRP-RRKPK
DmSoxB2b1     GHIKRPMNAF MVWSRLQRRQ IAKDNPKMHN SEISKRLGAE WKLLAESEKR PFIDEAKRLR ALHMKEHPDY KYRP-RRKPK
DmSoxB2b2     EHIKRPMNAF MVWSRLQRRK IAQDNPKMHN SEISKRLGAE WKLLTEEEKR PFIDEAKRLR AMHMKEHPDY KYRP-RRKPK
CeSoxB2       DHVKRPMNAF MVWSRGQRRK MAQDNPKMHN SEISKRLGAE WKQLSEQEKR PFIDEAKRLR ALHMKEHPDY KYRP-RRKPK
CspSoxB2      DHVKRPMNAF MVWSRGQRRK MAQENPKMHN SEISKRLGAE WKLLSEEEKR PFIDEAKRLR ALHMKDHPDY KYRP-RRKPK
LgSoxB2       DHVKRPMNAF MVWSRGQRRK MAQENPKMHN SEISKRLGAD WKLLTEEEKR PFIDEAKRLR ALHLKEHPDY KYRP-RRKPK
SkSoxB2       DHVKRPMNAF MVWSRGQRRK MAQENPKMHN SEISKRLGGE WKLLSEAEKR PFIDEAKRLR ALHMKEHPDY KYRP-RRKPK
SpSoxB2       DHVKRPMNAF MVWSRGQRRK LAQENPKMHN SEISKRLGAE WKLLSEDDKR PFIDEAKRLR ALHMKEHPDY KYRP-RRKPK
HsSox14       DHIKRPMNAF MVWSRGQRRK MAQENPKMHN SEISKRLGAE WKLLSEAEKR PYIDEAKRLR AQHMKEHPDY KYRP-RRKPK
HsSox21       DHVKRPMNAF MVWSRAQRRK MAQENPKMHN SEISKRLGAE WKLLTESEKR PFIDEAKRLR AMHMKEHPDY KYRP-RRKPK
NveSoxB1      DRVKRPMNAF MVWSRERRRK MAQDNPKMHN SEISKRLGSE WKLLSEQEKR PYIDEARRLR AVHMKEHPDY KYRP-RRKSK
NveSoxB2      GHVKRPMNAF MVWSRGKRKH YASINPRMHN SEISKRLGAE WKMLTAEEKE PFIAEAKRLQ ALHIQEHPDY KYKPKRRKPK
NveSox1       EHVKRPMNAF MVWSREERRK IAQENPKMHN SEISKRLGSE WKQLADDDKK PFVEEAKKLR AQHMKEHPDY KYRP-RRKPK
NveSox2       DHIKRPMNAY MVWSRKERRR IAEECPRMLN SEISKRLGLE WNSLTLDEKQ PYVEEAKRLR ELHKKDHPDY KYQP-KRKPK
NveSox3       NHVKRPMNAF MVWSKERRRI KSQECPRMHN SEISKILGCE WKATKDELKQ PYIEKAKELQ AQHSRENPGY KYKPRRRKPK
NveSoxA       QHIKRPMNAF MVWSRTERRK LALKYPNMLN CEISKLLGAE WSRLSEEEKR PFVTEAKRLR TIHNQKYPDY SYKPRRRKSK
AmiSoxB1      DRVKRPMNAF MVWSRERRRR MAQENPKMHN SEISKRLGAE WKQLSDPEKR PYVDEAKRLR AVHMKDHPDY KYRP-RRKSK
AmiSoxBa      GHIKRPMNAF MVWSRGKRKQ YAAINPRMHN SEISKRLGAE WKMLSQDEKE PFVAEAKRLQ AIHIQEHPDY KYKPKRRKPK
AmiSoxBb      DHIKRPMNAF MVWSKEKRRT MSQKNPKMHN SEISKILGAQ WKKMPDEEKA KYIEEAKRLQ QEHSQKHPDY KYKPRRRKQK
HmSoxB1       DKVKRPMNSF MVWSREKRRR LAHENPKMHN SEISKRLGAE WKVLTEDEKA PFVFEAKRLR AEHMKSHPDY KYRP-RRKAK
HmSoxBa       SHVKRPMNAF MVWSRGKRRQ MAQDNPRMHN SEISKRLGAE WKCLTQQEKQ PFIDEAKRLR AVHIQEHPDY KYKPKRRKQK
HmSoxBb       DHVKRPMNAF MVWSREKRRK MAQINPRMHN SEISKILGSE WKRMGESEKG PYVLEAKRLQ TQHSIEYPNY KYKPRRRKAK
HmSoxBc       DHVKRPMNAF MVWSRTERRK LALKYPNMLN CEISKLLGAE WSRMGEEEKS PYIQESKRLR TIHSQKYPDY SYKPRRRKRK
```
