# Supplementary figures and images for: Parallel Expansions of Sox Transcription Factor Group B Predating the Diversifications of the Arthropods and Jawed Vertebrates
Source: PLoS One. 2011 Jan 27;6(1):e16570. doi: 10.1371/journal.pone.0016570 (PMC3029401; doi:10.1371/journal.pone.0016570)

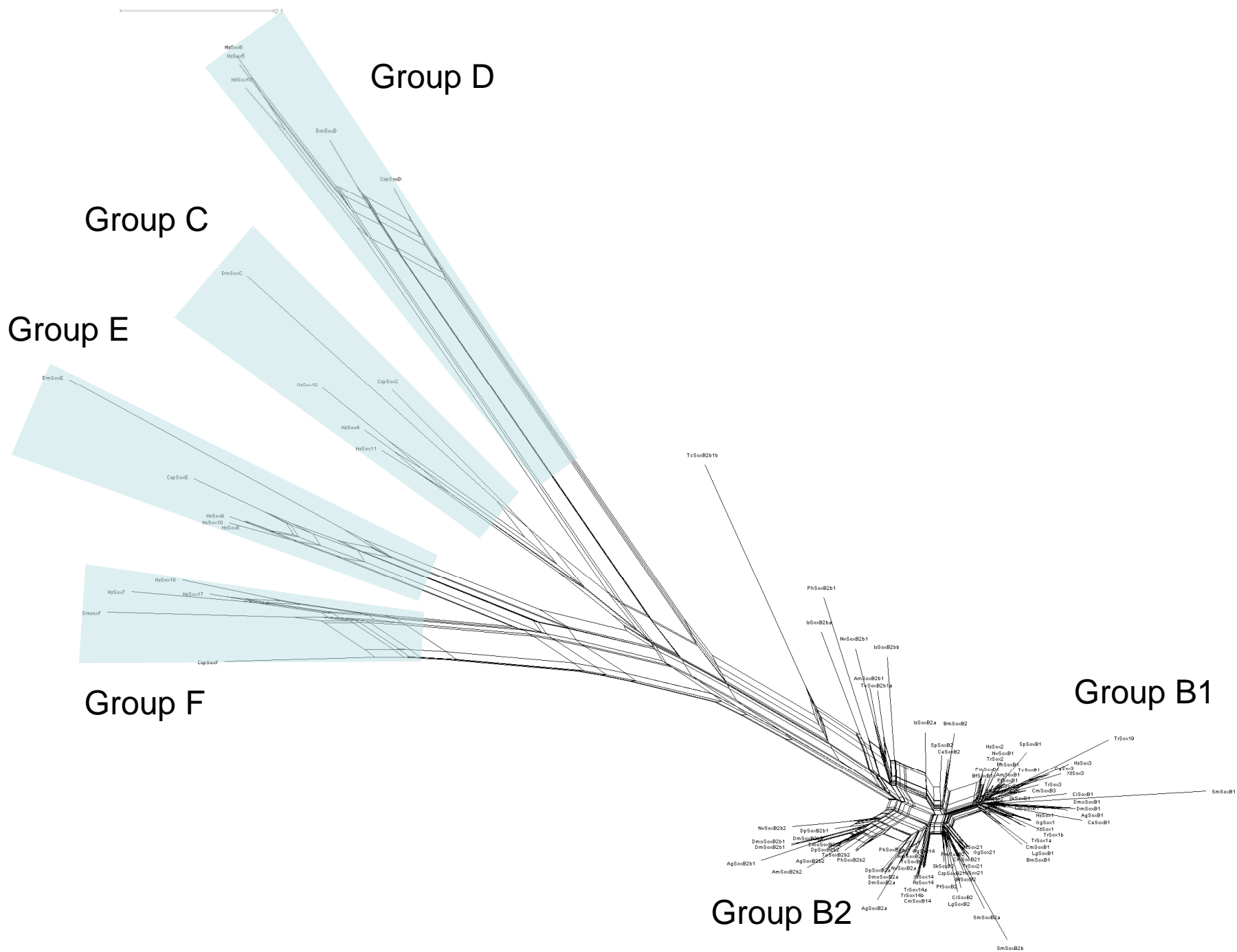

Supplement: Figure S3 — Split network of the bilaterian SoxB1/B2 proteins based on the HMG domain with sequences from Sox groups C, D, E, and F of human, Drosophila, and the annelid Capitella sp. I. The split network was reconstructed under the JTT model. Abbreviations of species names are as in Table 1. (PDF) [file pone.0016570.s003.pdf]

10.01

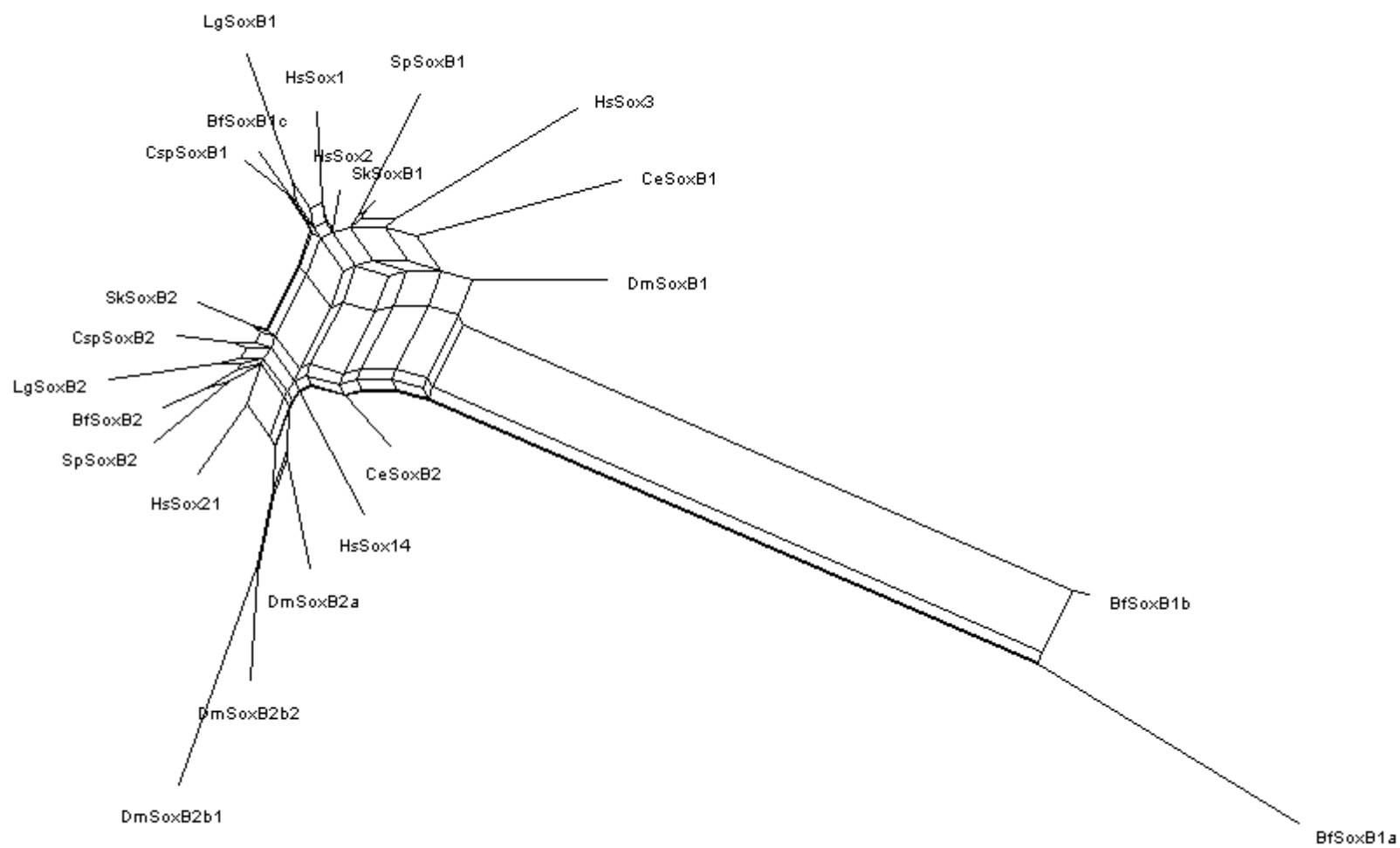

Supplement: Figure S4 — Split network of the HMG domain sequences of the SoxB1/B2 proteins in the full complements of Branchiostoma floridae and representative bilaterians, showing the signals of convergent evolution in BfSoxB1a and BfSoxB1b. The split network is based on the alignment shown in Fig. S2C. Abbreviations of species names are as in Table 1. (PDF) [file pone.0016570.s004.pdf]

A

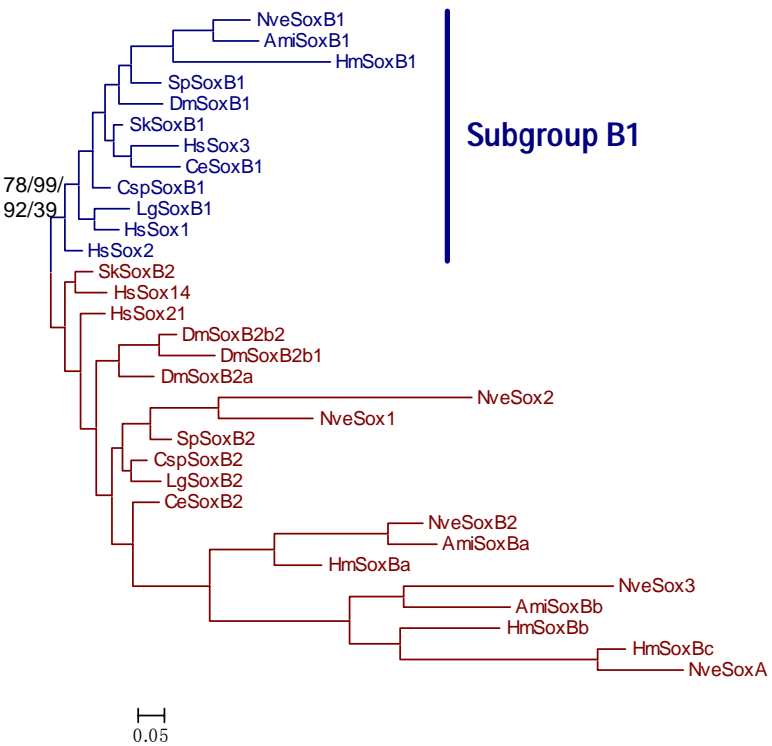

B

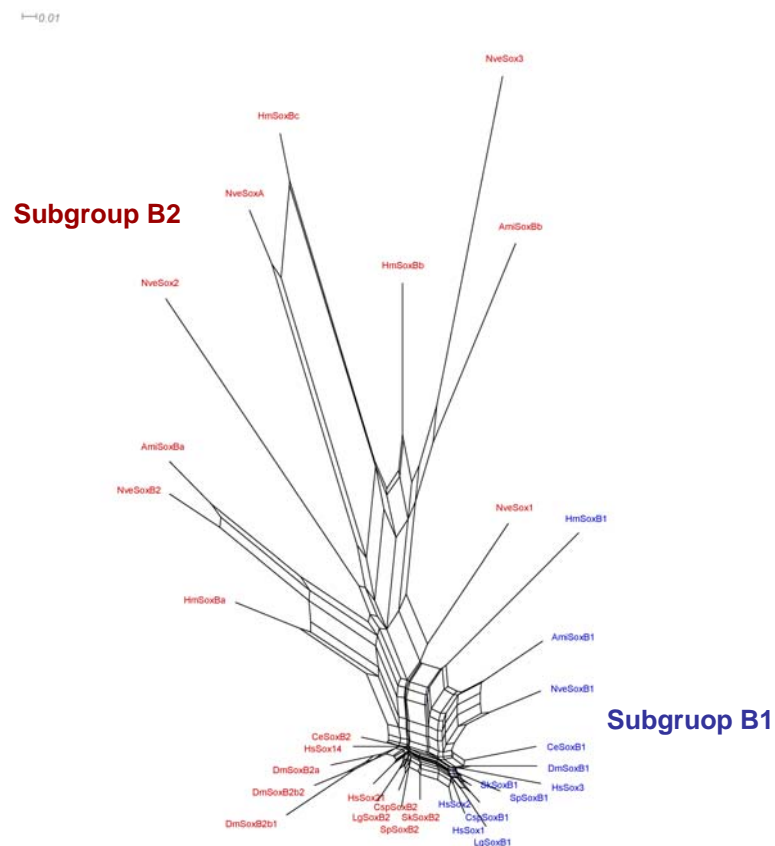

Supplement: Figure S5 — Phylogenetic tree and split network of the HMG domain sequences of the SoxB1/B2 proteins of three cnidarians and representative bilaterians. (A) Bayesian tree based on the alignment shown in Fig. S2D. Statistical support values for the SoxB1/SoxB2 split and the arthropod SoxB2b clade were derived with different methods, as described in Fig. 3. The model for the Bayesian reconstruction was RtREV + I + G; the model for the ML reconstruction was LG + I + G. (B) Split network under the JTT model is shown. Abbreviations of species names are as in Table 1. (PDF) [file pone.0016570.s005.pdf]

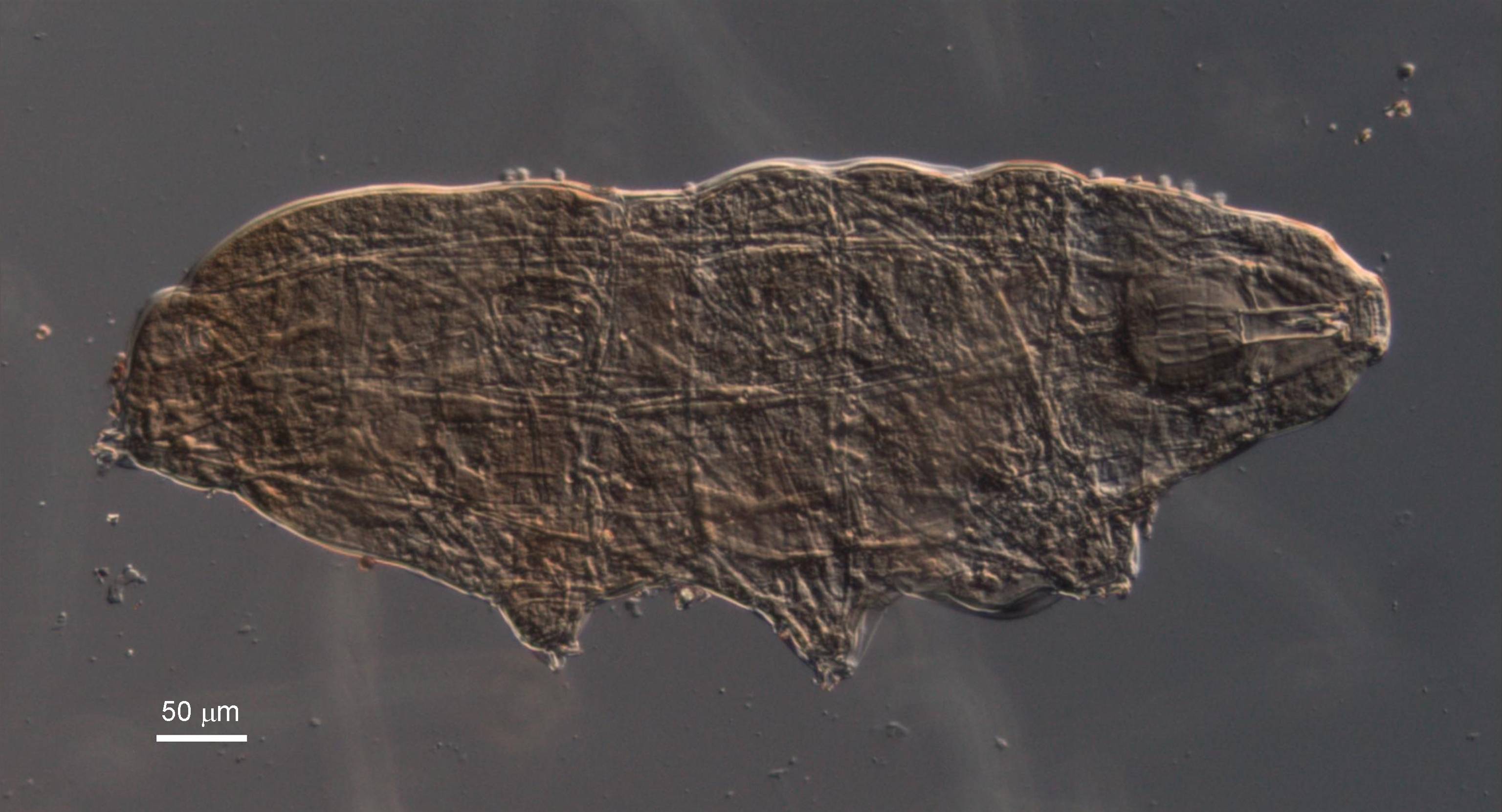

Supplement: Figure S6 — Photomicrograph of the whole body of the tardigrade Macrobiotus areolatus under a differential interference contrast microscope (DICM) with 100× magnification, from the mounting of Tong Yang. (JPG) [file pone.0016570.s006.jpg]

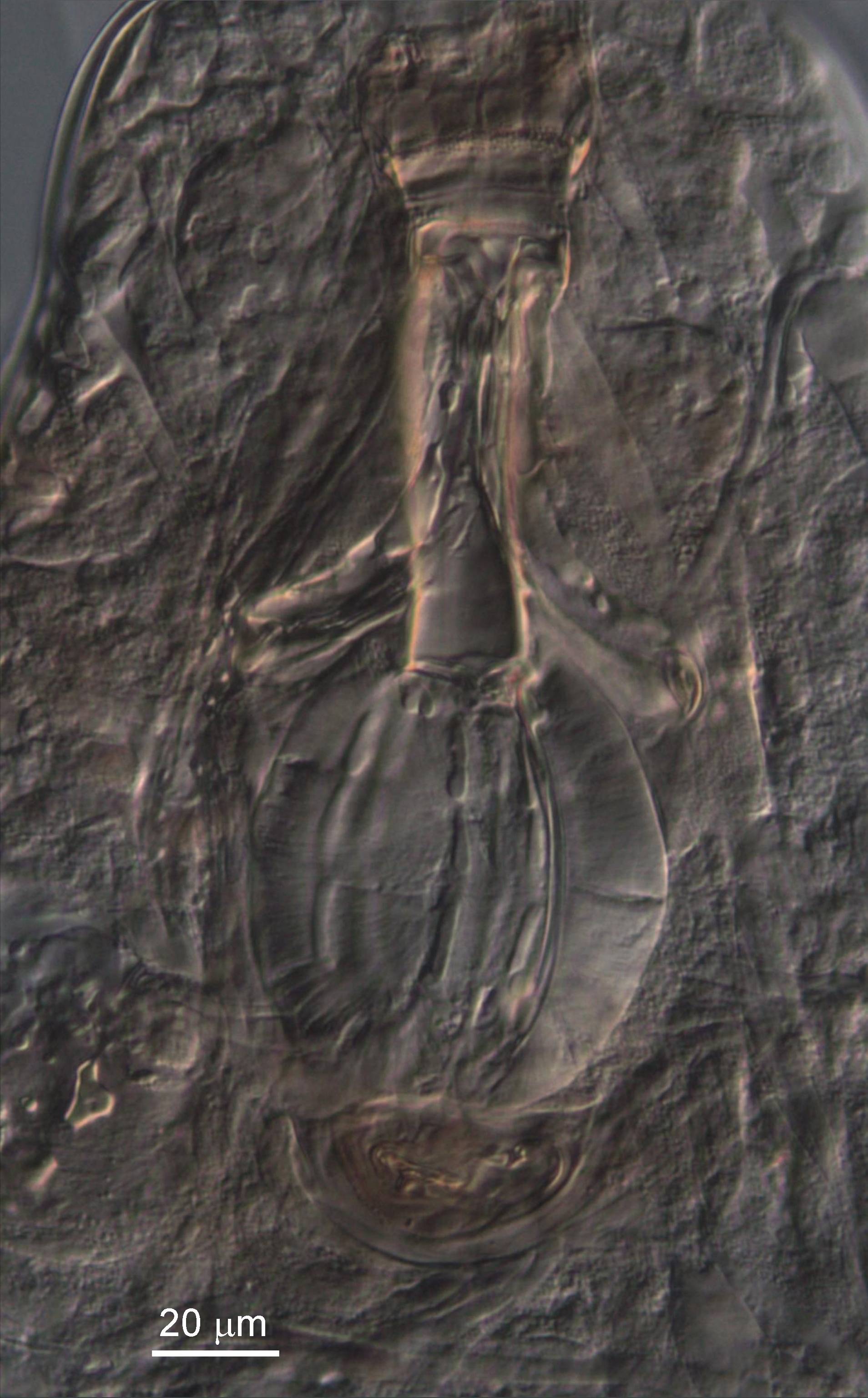

Supplement: Figure S7 — Photomicrograph of the pharynx of the tardigrade Macrobiotus areolatus under a DICM with 400× magnification, from the mounting of Tong Yang. (JPG) [file pone.0016570.s007.jpg]

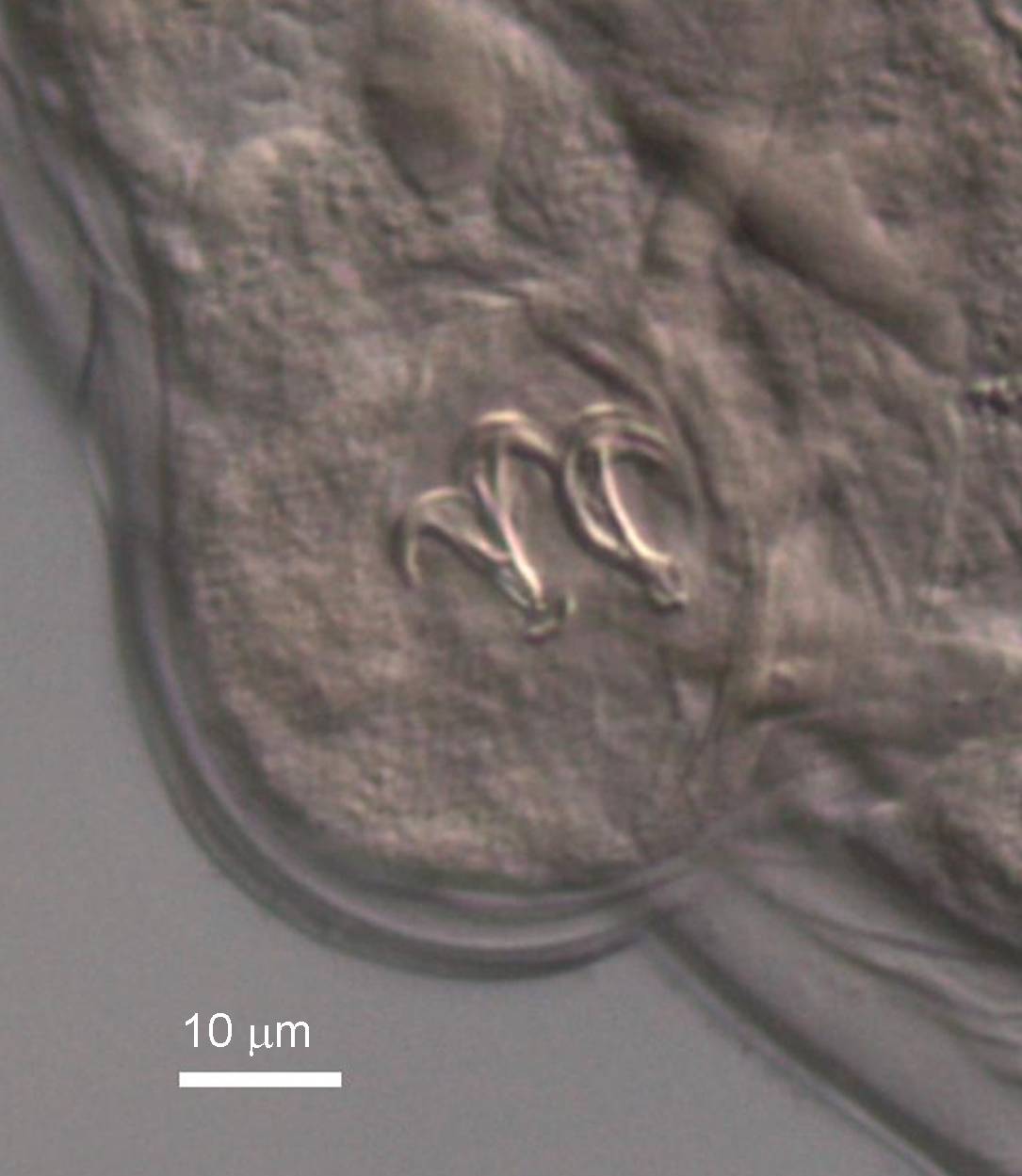

Supplement: Figure S8 — Photomicrograph of the claws of the tardigrade Macrobiotus areolatus under a DICM with 400× magnification, from the mounting of Tong Yang. (JPG) [file pone.0016570.s008.jpg]
